# An analytical study of sound transmission loss of functionally graded sandwich cylindrical nanoshell integrated with piezoelectric layers

**DOI:** 10.1038/s41598-022-06905-1

**Published:** 2022-02-23

**Authors:** Chanachai Thongchom, Pouyan Roodgar Saffari, Nima Refahati, Peyman Roudgar Saffari, Hossein Pourbashash, Sayan Sirimontree, Suraparb Keawsawasvong

**Affiliations:** 1 Department of Civil Engineering, Faculty of Engineering, Thammasat School of Engineering, Thammasat University, Pathumthani, Thailand; 2 Department of Mechanical Engineering, Damavand Branch, Islamic Azad University, Damavand, Iran; 3 Department of Mathematics, University of Garmsar, Garmsar, Iran

**Keywords:** Engineering, Mechanical engineering

## Abstract

The multidisciplinary nature of piezoelectric (PZ) structures necessitates precise and efficient methods to express their behavior under different conditions. This article extends the general usage of PZ materials by introducing acoustic and fluid loading effects in a way that an unfilled multilayer cylindrical nanoshell with a functionally graded (FG) material core and PZ layers is subjected to preliminary external electric load, acoustic waves and external flow motion. As the properties of a functionally graded material changes along the shell thickness, a power law model is assumed to be governing such variations of desired characteristics. Evidently, this system includes different types of couplings and a comprehensive approach is required to describe the structural response. To this aim, the first-order shear deformation theory (FSDT) is used to define different displacement components. Next, the coupled size-dependent vibroacoustic equations are derived based on in conjunction with nonlocal strain gradient theory (NSGT) with the aid of Hamilton’s variational principle and fluid/structure compatibility conditions. NSGT is complemented with hardening and softening material effects which can greatly enhance the precision of results. It is expected to use the findings of this paper in the optimization of similar systems by selecting suitable FG index, incident angle of sound waves, flow Mach number, nonlocal and strain gradient parameters, starting electric potential and geometric features. One of the important findings of this study is that increasing the electric voltage can obtain better sound insulation at small frequencies, specially prior to the ring frequency.

## Introduction

Nowadays, there is hardly any industry where thin cylindrical shells cannot be found. It is no secret that such thin structures are prone to different types of vibrations, and mitigating them is of great concern to engineers and technicians. These vibrations are closely associated with acoustic problems, and one area where near-field acoustic radiation must be carefully considered is concealing the whole system from various radar technologies^1–5^. When a system benefiting from cylindrical shells is susceptible to any type of dynamic loads, a complete evaluation of its vibration behavior is indispensable. There is a rich collection of analytical, numerical and experimental studies targeting the vibration of various types of shells using different simplifying assumptions^6–9^.

The concept of PZ has been extensively addressed in electrical and mechanical engineering. Many devices have been built around these materials, either using their direct or reverse effects so that they can be used as sensors or actuators, respectively^10–12^. As an inorganic compound, lead zirconate titanate constitutes a common type of PZ ceramics^13,14^. These special ceramics are also used in the production of PZ sensors (patches)^15,16^ and actuators (stacks)^17,18^. Almost as famous as PZ ceramics, PZ polymers^19,20^ comprise another commonplace category of PZ materials with specific advantages. Polyvinylidene difluoride (PVDF) is a well-known PZ polymer that offers flexibility and lightness unlike most PZ ceramics^21,22^. Sheng and Wang^23^ presented buckling and thermoelastic vibration characteristics of the FG PZ cylindrical subjected to the initial external electric voltage. Xu et al.^24^ analyzed the coupled vibration of a radially polarized PZ cylindrical transducer using mechanical coupling coefficient method. Bisheh and Wu^25^ investigated the wave propagation problem of PZ cylindrical composite shells reinforced with carbon nanotubes utilizing FSDT and Mori–Tanaka method. Li et al.^26^ suggested a smart model for the vibration control of discontinuous PZ laminated shell with point supported elastic boundary conditions based FSDT and the Chebyshev polynomial. The nonlinear dynamic response of fluid-conveying FG cylindrical shells with PZ actuator layer is presented by Wang et al.^27^ based on von-Karman geometrical nonlinearity. Li et al.^28^ applied FSDT to vibration suppression of laminated cylindrical shells with discontinuous PZ layer with the negative velocity feedback adjustment.

Functionally graded materials (FGMs), similar to many other types of composites, provide superior performance in comparison with homogeneous materials by combining the required properties of each constituent phase^29–37^. This advantage in the case of FGMs is realized by the gradual changeover of components/microstructures including porosity and texture in one or more directions. It is thus expected to see a notable change in one or more properties. The continuous and smooth variations of different mechanical and thermal properties give FGMs a great power for implementation in applications where uniform characteristics are not desirable. Ghadiri and Safarpour^38^ employed the FSDT to examine the thermo-mechanical vibration response of FG microshell with porosity. They assumed that according to a power-law model, the remarkable material properties are associated with the porosity volume fraction and are considered to be constantly changeable through the thickness direction. Ninh et al.^39^ calculated the dynamic response of the conveying-fluid toroidal shell segments made of FG graphene nanoplatelets with PZ layers. But they did not study the effect of initial electric voltage on the variation of dynamic response in the frequency range. A wave-based is proposed by Liu et al.^40^ to analytically determine the free vibration properties of FGM cylindrical shells with arbitrary boundary conditions (i.e., both elastic support boundary conditions and classical boundary conditions) based on FSDT. Sofiyev^41^ presented an analytical method to study the dynamic behavior of the infinitely-long FGM cylindrical shell under moving loads. Ye and Wang^42^ obtained the nonlinear dynamical response of cylindrical shells reinforced with FG graphene platelets using Donnell’s nonlinear shell theory. Belabed et al.^43^ employed an efficient higher order shear deformation theory to investigate the natyral frequencies of FG shells.

Nanotechnology is the use of any matter at very small scales. By doing so, one can gain exceptional benefits that would otherwise be impossible to achieve at macroscale. Today, most engineering fields have considered the use of nanomaterials for different purposes. Understanding the mechanical response of nanostructures is the key to their successful implementation in different applications. Despite the popularity of classic continuum theories, they simply fail at such small scales^44–47^. The purpose of nonlocal continuum theories is to fill this gap. In addition, non-classical continuum theories are notably useful and practical compared to atomistic models. They are also employed to deal with such small-scale phenomena. This class of theories have many branches, some of which include the nonlocal elasticity theory^48,49^, modified couple stress theory^50,51^, modified strain gradient theory^52–56^, and NSGT^57–60^. So far, these nonclassical theories have been used by some researchers to predict small scale size effect of macro/nanostructures. For example, Liu et al.^61^ discussed the influence of NSGT on nonlinear dynamic of FG multilayer beam-type nanostructures reinforced by graphene nanoplatelet considering the initial geometric imperfection. Based on the NSGT in conjunction with FSDT, Liu and Lyu^62^ presented the theoretical modeling for investigating the frequency shift behavior of nano-mass sensor system composed of smart core integrated with graphene layers. Zhang and Liu^63^ used modified couple stress theory and power-law distribution form to study the dynamic behavior of FG microbeams with different porosity distributions under moving harmonic load. The nonlocal theory and Love’s thin shell are carried out by Ke et al.^64^ to investigate thermo-electro-mechanical free vibration of PZ cylindrical nanoshells. One of the drawbacks of this study is that the authors did not consider the shear effect on the dynamic behavior of the proposed system. Mohammadi et al.^65^ used NSGT in conjunction with FSDT to study the natural frequencies of FG nanoshells. Ebrahimi-Mamaghani et al.^66^ investigated the vibration reduction in piping structures attached to a nonlinear absorber. They determined the dynamical response and stability threshold of the considered system. Also, they demonstrated that nonlinear absorber has an appropriate efficiency in the vibration mitigation of pipes. Saffari et al.^67^ The literature is study dealing with the effects of small-scale phenomena on the free and force vibrations as well as static and dynamic stability problems of plates, and BNNTs. Zarabimanesh et al.^68^ investigated the size-dependent free vibration of two vertically aligned single-walled boron nitride nanotubes conveying fluid subjected to hygrothermal environment using NSGT.

The transmission of sound waves after hitting an object has been of notable significance for acoustic and mechanical engineers. So far, analytical relationships have been obtained for almost any practical structure (for example multi-layer plates) in any viable scenario (such as submarines and concrete walls). However, most analytical solutions pose serious difficulties when trying to obtain their exact solution. To tackle this issue, approximate approaches for acoustic problems have gained popularity in recent years. Heckl^69^ gave a thorough account of sound transmission through different types of building walls almost a century ago. A review of acoustic estimation methods has been published by Pellicier and Trompette^70^, and gives readers valuable information on the wave approach. Sound transmission loss (STL) is a quantitative description of how a structure attenuates the incident sound waves, and is thus a quantity of interest in noise control applications^71–79^. Yang et al.^80^ proposed the extension of a wave and finite element (WFE) method for predicting sound radiation and transmission characteristics of infinite panels. Kingan et al.^81^ analyzed sound transmission through, and radiation from, an infinitely long cylindrical structure using the WFE. Lee and Kim^82^ analytically and experimentally analyzed the properties of STL across a cylindrical shell using classical shell theory. Daneshjou et al.^83^ presented an analytical method for predicting the STL across thick cylindrical shells made of FGMs using third order shear deformation theory. Golzari and Jafari^84^ used Biot's theory for modeling porous material in triple-walled sandwich cylindrical shells. The main results of their work denoted a better efficiency in the noise improvement for the triple-walled cylindrical shell, noticeably at mid-high and high frequencies, in comparison with its double-walled cylindrical shell. Heydari et al.^85^ studied the STL of cylindrical FG nanoshell considering porous materials using NSGT in conjunction with FSDT. One of the drawbacks of these studies is that the simultaneous effects of small scale and electric voltage of piezoelectric layers on the STL behavior of cylindrical shells have not been investigated. Nowadays, it is no secret that nanoscience is critically important in various fields of engineering, medicine and treatment of diseases, in particular cancer. Cylindrical nanoshells constitute a type of nanostructures with numerous applications in the field of drug delivery in the body thanks to the acceptable balance between their structural weight and mechanical strength. Nanoshells are exposed to sound waves in fluids, resulting in fatigue and cracking over time. Hence, investigating the effects of acoustic waves on the dynamic behavior of nanoshells is of considerable importance.

In light of the presented literature review and the lack of any comprehensive study on the sound transmission/wave propagation in FG-PZ cylindrical nanoshells, this article first obtains the relationships of STL for a sandwich cylindrical nanoshell benefiting from FGMs and PZ layers when exposed to outer flow in certain velocity ranges using NSGT and FSDT. Based on the power law developed for FGMs, the properties of core layer vary in the thickness direction, while imposing proper boundary conditions between the structure and encompassing medium allows us to capture acoustic effects.

## Theoretical formulations

**Figure 1:**
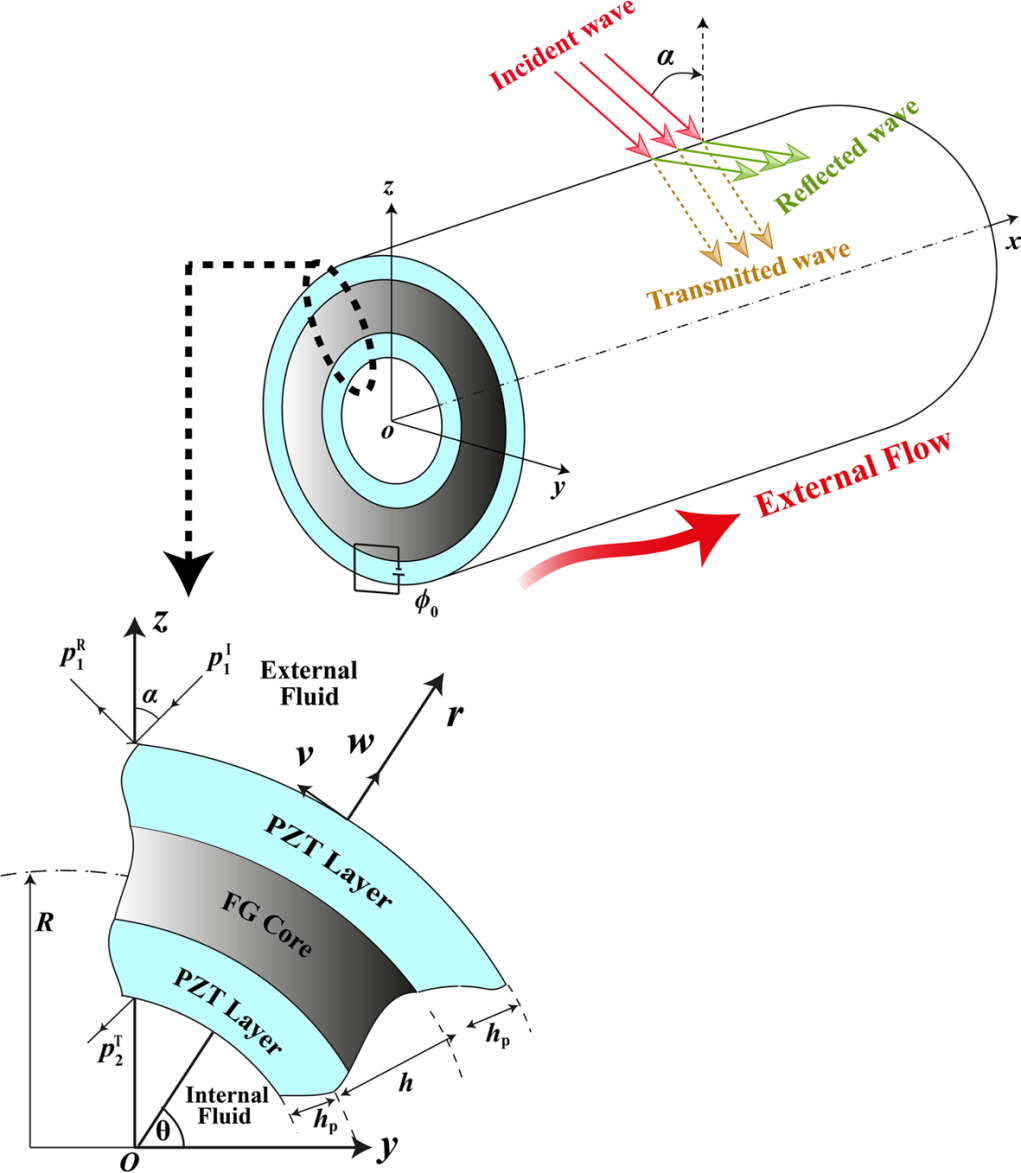
The schematic of a sandwich FG-PZ cylindrical nanoshell under incidence wave.

The Fig. 1 was drawn by Pouyan Roodgar Saffari in which sound waves hit the sandwich cylindrical shell at the incident angle $0<\alpha <\pi /2$. Both the exterior and interior of the sandwich cylindrical shell are filled with air of the characteristic impedance $(\rho , c)$. The sandwich cylindrical shell includes an FG core (including a ceramic phase and a metal phase) of thickness $h$ and radius $R$ while being surrounded by external and internal piezoelectric layers of thickness ${h}_{p}$. Noteworthy is that the outer piezoelectric layer is treated as an actuator with a specific input voltage ${\phi }_{0}$, while the internal piezoelectric layer is modeled as a sensor. In addition, a steady flow of air passes over the structure at the velocity $V$.

### Acoustic field equations

A proper approach to deal with the system at hand is to consider two distinct incident and transmitted fields, with the incident ${p}_{1}^{\mathrm{I}}$ and reflected ${p}_{1}^{\mathrm{R}}$ waves satisfying the wave equation in the external medium as^86^

1 $${c}^{2}{\nabla }^{2}\left({p}_{1}^{\mathrm{I}}+{p}_{1}^{\mathrm{R}}\right)+{\left(\frac{\partial }{\partial t}+V \cdot \nabla \right)}^{2}\left({p}_{1}^{\mathrm{I}}+{p}_{1}^{\mathrm{R}}\right)=0,$$

where the Laplace operator in the cylindrical coordinates signified with ${\nabla }^{2}=\frac{1}{r}\frac{\partial }{\partial r}\left(r\frac{\partial }{\partial r}\right)+\frac{1}{{r}^{2}}\frac{{\partial }^{2}}{\partial {\theta }^{2}}+\frac{{\partial }^{2}}{\partial {x}^{2}}$. Furthermore, the external flow velocity is indicated with *V*. Concerning the interior of the structure, the transmitted wave ${p}_{2}^{\mathrm{T}}$ is all that exists. Consequently, the wave equation of this anechoic cavity expresses as

2 $${c}^{2}{\nabla }^{2}{p}_{2}^{\mathrm{T}}=\frac{{\partial }^{2}}{\partial {t}^{2}}{p}_{2}^{\mathrm{T}},$$

For cylindrical coordinate system, the terms of time-harmonic pressure waves are defined as^86^

3 $$\begin{aligned} {p}_{1}^{\mathrm{I}}\left(r,\theta ,x,t\right) & ={p}_{0}{e}^{\mathrm{i}\left(\omega t-{k}_{x}x\right)}\sum_{n=0}^{\infty }{\varepsilon }_{n}{\left(-\mathrm{i}\right)}^{n}{J}_{n}\left({k}_{1r}r\right)\mathrm{cos}\left(n\theta \right), \\ {p}_{1}^{\mathrm{R}}\left(r,\theta ,x,t\right) & ={e}^{\mathrm{i}\left(\omega t-{k}_{x}x\right)}\sum_{n=0}^{\infty }{\tilde{P }}_{1n}^{\mathrm{R}}{H}_{n}^{\left(2\right)}\left({k}_{1r}r\right)\mathrm{cos}\left(n\theta \right), \\ {p}_{2}^{\mathrm{T}}\left(r,\theta ,x,t\right) & ={e}^{\mathrm{i}\left(\omega t-{k}_{x}x\right)}\sum_{n=0}^{\infty }{\tilde{P }}_{2n}^{\mathrm{T}}{H}_{n}^{\left(1\right)}\left({k}_{3r}r\right)\mathrm{cos}\left(n\theta \right). \end{aligned}$$

where ${p}_{0}$ refers to the amplitude of pressure of the incident wave, $\omega$ shows the angular frequency, ${\varepsilon }_{0}=1, {\varepsilon }_{n}=2(n\ge 1)$, $\mathrm{i}=\sqrt{-1}, { J}_{n}$ denotes the cylindrical Bessel function of the first kind and n-th order, ${H}_{n}^{\left(1\right)}$ and ${H}_{n}^{\left(2\right)}$ signify, respectively, the cylindrical Hankel functions of the first and second kinds. Additionally, $({\tilde{P }}_{1n}^{\mathrm{R}},{\tilde{P }}_{2n}^{\mathrm{T}}, {\tilde{P }}_{2n}^{\mathrm{R}}, {\tilde{P }}_{3n}^{\mathrm{T}})$ are unknown complex coefficients. Furthermore, the radial and axial components of the wavenumbers are stated as

4 $$\begin{aligned}{k}_{x} & ={k}_{1}\mathrm{sin}\alpha , {k}_{1r}={k}_{1}\mathrm{cos}\alpha ={\left[{k}_{1}^{2}-{k}_{x}^{2}\right]}^{1/2}, \\ {k}_{2r} & =\sqrt{{k}_{2}^{2}-{k}_{x}^{2}}, {k}_{1}=\omega /\left[c\left(1+M\mathrm{sin}\alpha \right)\right], {k}_{2}=\omega /c,\end{aligned}$$

where $M=V/c$ is the Mach number of the external flow.

### Structural field equations

There are a number of conflicts in the FSDT which could grow to be vital in certain thick laminates or sandwich systems that possess a small transverse shear modulus. To compensate the lack of varying transverse shear strains along the thickness in this theory, substitute transverse shear strains are assumed on the laminate surfaces, although such stresses are zero in reality. However, different components of displacement are expressed as^66^

5 $$\begin{aligned}U\left(x,\theta ,z,t\right) & =u\left(x,\theta ,t\right)+z{\psi }_{x}\left(x,\theta ,t\right),\\ V\left(x,\theta ,z,t\right) & =v\left(x,\theta ,t\right)+z{\psi }_{\theta }\left(x,\theta ,t\right),\\ W\left(x,\theta ,z,t\right) & =w\left(x,\theta ,t\right),\end{aligned}$$

in which the in-plane deflections of the mid-surface along $x$ and $\theta$ directions are denoted with $u$ and $v$, respectively, and $w$ is the transverse deflection of the nanoshell in z-direction. Furthermore, the rotation angles of the middle plane along $\theta$ and $x$ directions signify, respectively, with ${\psi }_{\theta }$ and ${\psi }_{x}$. The expanded form of displacements and rotations are expressed as^87,88^

6 $$\begin{aligned}\langle u,w,{\psi }_{x}\rangle & =\sum_{n=0}^{\infty }{e}^{\mathrm{i}\left({\omega t-k}_{x}x\right)}\langle \tilde{u },\tilde{w },{\stackrel{\sim }{\psi }}_{x}\rangle \mathrm{cos}\left(n\theta \right), \\ \langle v,{\psi }_{\theta }\rangle & =\sum_{n=0}^{\infty }{e}^{\mathrm{i}\left({\omega t-k}_{x}x\right)}\langle {v}_{1},{\stackrel{\sim }{\psi }}_{\theta }\rangle \mathrm{sin}\left(n\theta \right)\end{aligned}$$

where $\langle \tilde{u },{\stackrel{\sim }{\psi }}_{x},\tilde{v },{\stackrel{\sim }{\psi }}_{\theta },\tilde{w }\rangle$ are the unknown modal coefficients. Based on the concept of FSDT, the relationships between different strain and displacement/rotation components for a sandwich FG-PZ cylindrical shell are presented as

7 $$\begin{aligned} {\varepsilon }_{xx} & =\frac{\partial u}{\partial x}+z\frac{\partial {\psi }_{x}}{\partial x}, \\ {\varepsilon }_{\theta \theta } & =\frac{1}{R}\frac{\partial v}{\partial \theta }+\frac{z}{R}\frac{\partial {\psi }_{\theta }}{\partial \theta }+\frac{w}{R}, \\ {\gamma }_{x\theta } & =\frac{\partial v}{\partial x}+\frac{1}{R}\frac{\partial u}{\partial \theta }+z\left(\frac{1}{R}\frac{\partial {\psi }_{x}}{\partial \theta }+\frac{\partial {\psi }_{\theta }}{\partial x}\right), \\ {\gamma }_{\theta z} &={\psi }_{\theta }+\frac{1}{R}\frac{\partial w}{\partial \theta }-\frac{v}{R}, \\ {\gamma }_{xz}& =\frac{\partial w}{\partial x}+{\psi }_{x}. \end{aligned}$$

where $\left({\gamma }_{x\theta }, {\gamma }_{xz},{\gamma }_{\theta z}\right)$ refer to the shear strains and $\left({\varepsilon }_{xx}, {\varepsilon }_{\theta \theta }\right)$ are the normal strain components. As referred to earlier in the article, the core of sandwich structure is made from FGM comprised of metal and ceramic. This article relies on a power law model with the following description

8 $$\begin{aligned} E\left(z\right) &={E}_{m}+\left({E}_{c}-{E}_{m}\right){\left(1/2+z/h\right)}^{\lambda }, \\ \rho \left(z\right) & ={\rho }_{m}+\left({\rho }_{c}-{\rho }_{m}\right){\left(1/2+z/h\right)}^{\lambda }, \\ \vartheta \left(z\right) &={\vartheta }_{m}+\left({\vartheta }_{c}-{\vartheta }_{m}\right){\left(1/2+z/h\right)}^{\lambda }, \end{aligned}$$

where $m$ and $c$ signify, respectively, metal and ceramic phases. Furthermore, the Young's modulus, mass density, Poisson’s ratio, respectively, specify with $E$, $\rho$, $\vartheta$. The always-positive gradient index ($\lambda )$ is used in this study to determine the changes of a specific property in the thickness direction. The greater the gradient index, the more metallic the structure. Thus, an isotropic ceramic is obtained by assuming a very small gradient index. Based on NSGT, the nonclassical constituent relations between stress and strain tensors for heterogeneous core layer are presented as^89^

9 $$\left[1-{\left({e}_{0}a\right)}^{2}{\nabla }^{2}\right]\left[\begin{array}{c}\begin{array}{c}\begin{array}{c}{\sigma }_{xx}^{\mathrm{FG}}\\ {\sigma }_{\theta \theta }^{\mathrm{FG}}\end{array}\\ {\tau }_{x\theta }^{\mathrm{FG}}\end{array}\\ {\tau }_{\theta z}^{\mathrm{FG}}\\ {\tau }_{xz}^{\mathrm{FG}}\end{array}\right]=\left[1-{l}^{2}{\nabla }^{2}\right]\left\{\begin{array}{ccccc}\frac{E\left(z\right)}{1-{\vartheta \left(z\right)}^{2}}& \frac{\vartheta \left(z\right)E\left(z\right)}{1-{\vartheta \left(z\right)}^{2}}& 0& 0& 0\\ \frac{\vartheta \left(z\right)E\left(z\right)}{1-{\vartheta \left(z\right)}^{2}}& \frac{E\left(z\right)}{1-{\vartheta \left(z\right)}^{2}}& 0& 0& 0\\ 0& 0& \frac{E\left(z\right)}{2\left(1+\vartheta \left(z\right)\right)}& 0& 0\\ 0& 0& 0& \frac{E\left(z\right)}{2\left(1+\vartheta \left(z\right)\right)}& 0\\ 0& 0& 0& 0& \frac{E\left(z\right)}{2\left(1+\vartheta \left(z\right)\right)}\end{array}\right\}\left[\begin{array}{c}\begin{array}{c}\begin{array}{c}{\varepsilon }_{xx}\\ {\varepsilon }_{\theta \theta }\end{array}\\ {\gamma }_{x\theta }\end{array}\\ {\gamma }_{\theta z}\\ {\gamma }_{xz}\end{array}\right],$$

where the terms $l$ and ${e}_{0}a$ signify the strain gradient and nonlocal parameter, respectively. ${e}_{0}$ refers to the calibration constant and $a$ denotes the internal characteristic length. The polling direction for the considered piezoelectric material lies along the positive z-axis. According to the NSGT, the general constitutive equations for inner and outer PZ layers can be presented as^90,91^

10 $$\begin{aligned}\left[1-{\left({e}_{0}a\right)}^{2}{\nabla }^{2}\right]\left[\begin{array}{c}\begin{array}{c}\begin{array}{c}{\sigma }_{xxi}^{PZT}\\ {\sigma }_{\theta \theta i}^{PZT}\end{array}\\ {\tau }_{x\theta i}^{\mathrm{PZ}}\end{array}\\ {\tau }_{\theta zi}^{\mathrm{PZ}}\\ {\tau }_{xzi}^{\mathrm{PZ}}\end{array}\right] & =\left[1-{l}^{2}{\nabla }^{2}\right]\left(\left\{\begin{array}{ccccc}{c}_{11}& {c}_{12}& 0& 0& 0\\ {c}_{12}& {c}_{22}& 0& 0& 0\\ 0& 0& {c}_{66}& 0& 0\\ 0& 0& 0& {c}_{44}& 0\\ 0& 0& 0& 0& {c}_{55}\end{array}\right\}\left[\begin{array}{c}\begin{array}{c}\begin{array}{c}{\varepsilon }_{xx}\\ {\varepsilon }_{\theta \theta }\end{array}\\ {\gamma }_{x\theta }\end{array}\\ {\gamma }_{\theta z}\\ {\gamma }_{xz}\end{array}\right]-\left\{\begin{array}{ccc}0& 0& {e}_{31}\\ 0& 0& {e}_{32}\\ 0& 0& 0\\ 0& {e}_{24}& 0\\ {e}_{15}& 0& 0\end{array}\right\}\left[\begin{array}{c}{\mathcal{F}}_{xi}\\ {\mathcal{F}}_{\theta i}\\ {\mathcal{F}}_{zi}\end{array}\right]\right), \\ \left[1-{\left({e}_{0}a\right)}^{2}{\nabla }^{2}\right]\left[\begin{array}{c}\begin{array}{c}{\mathcal{D}}_{xi}\\ {\mathcal{D}}_{\theta i}\end{array}\\ {\mathcal{D}}_{zi}\end{array}\right] & =\left[1-{l}^{2}{\nabla }^{2}\right]\left(\left\{\begin{array}{ccccc}0& 0& 0& 0& {e}_{15}\\ 0& 0& 0& {e}_{24}& 0\\ {e}_{31}& {e}_{32}& 0& 0& 0\end{array}\right\}\left[\begin{array}{c}\begin{array}{c}\begin{array}{c}{\varepsilon }_{xx}\\ {\varepsilon }_{\theta \theta }\end{array}\\ {\gamma }_{x\theta }\end{array}\\ {\gamma }_{\theta z}\\ {\gamma }_{xz}\end{array}\right]+\left\{\begin{array}{ccc}{k}_{11}& 0& 0\\ 0& {k}_{22}& 0\\ 0& 0& {k}_{33}\end{array}\right\}\left[\begin{array}{c}{\mathcal{F}}_{xi}\\ {\mathcal{F}}_{\theta i}\\ {\mathcal{F}}_{zi}\end{array}\right]\right), i=\mathrm{ex},\mathrm{in}. \end{aligned}$$

where ${c}_{66}=\frac{{c}_{11}-{c}_{12}}{2}$ and the internal and external PZ layers are expressed with $\mathrm{in}$ and $\mathrm{ex}$. Also, term $\left[\mathcal{D}\right]$ expresses, the electric displacement. Furthermore, $\left[{\varvec{\kappa}}\right], \left[{\varvec{e}}\right]$, and $\left[{\varvec{c}}\right]$ denote the dielectric, piezoelectric, and the elastic constant matrices, respectively. Moreover, $\left[\mathcal{F}\right]$ represents the electric field. A common method for expressing the changes of different electric ($\Phi$) potential in the thickness direction of internal and external PZ layers, as described in several previous studies, is presented as^92^

11 $$\begin{aligned}{\Phi }_{\mathrm{ex}}\left(x,\theta ,z,t\right) & =\left[{\left(z-\frac{h+{h}_{p}}{2}\right)}^{2}-{\left(\frac{{h}_{p}}{2}\right)}^{2}\right]{\phi }_{\mathrm{ex}}\left(x,\theta ,t\right)+2\left(z-\frac{h+{h}_{p}}{2}\right){\phi }_{0}, \\ {\Phi }_{\mathrm{in}}\left(x,\theta ,z,t\right) & =\left[{\left(z+\frac{h+{h}_{p}}{2}\right)}^{2}-{\left(\frac{{h}_{p}}{2}\right)}^{2}\right]{\phi }_{\mathrm{in}}\left(x,\theta ,t\right),\end{aligned}$$

in which $\phi$ denotes the two-dimensional electric potential of external and internal PZ layers. However, the expanded forms of electric potential can be presented as

12 $$\langle {\phi }_{in},{\phi }_{ex}\rangle =\sum_{n=0}^{\infty }{e}^{\mathrm{i}\left({\omega t-k}_{x}x\right)}\langle {\stackrel{\sim }{\phi }}_{in},{\stackrel{\sim }{\phi }}_{ex}\rangle \mathrm{cos}\left(n\theta \right),$$

To satisfy Maxwell’s equations in the proposed procedure, two assumptions are made: the electric field presents as the negative gradient of $\Phi$. Accordingly, one can write^92^

13 $${\mathcal{F}}_{x}=-\frac{\partial }{\partial x}\Phi , {\mathcal{F}}_{\theta }=-\frac{1}{R+z}\frac{\partial }{\partial \theta }\left(\Phi ,\Psi \right), {\mathcal{F}}_{z}=-\frac{\partial }{\partial z}\Phi$$

However, in order to obtain the vibroacoustic equations, one needs to carefully implement Hamilton’s principle and its different dependencies to be able to arrive at equilibrium equations of motion. This procedure is stated as

14 $${\int }_{0}^{t}\updelta \left({\Gamma }_{\mathrm{s}}+{\Gamma }_{\mathrm{f}}-{\Gamma }_{\mathrm{K}}\right)\mathrm{d}t=0,$$

where (${\Gamma }_{\mathrm{K}}, {{\Gamma }_{\mathrm{f}},\Gamma }_{\mathrm{s}}$) indicate the kinetic energy per unit volume of the cylindrical nanoshell, the work done by external forces (the work applied by the incidence sound wave) per unit area, and strain energy per unit volume. However, the variation of kinetic energy for the system is expressed based on the FSDT as

15 $$\begin{aligned}\delta {\Gamma }_{\mathrm{K}}&={\int }_{A}{\int }_{-\frac{h}{2}-{h}_{p}}^{-\frac{h}{2}}{\rho }_{PZT}\left(\dot{U}\delta \dot{U}+\dot{V}\delta \dot{V}+\dot{W}\delta \dot{W}\right)\mathrm{d}z\mathrm{dA}+{\int }_{A}{\int }_{-\frac{h}{2}}^\frac{h}{2}\rho \left(z\right)\left(\dot{U}\delta \dot{U}+\dot{V}\delta \dot{V}+\dot{W}\delta \dot{W}\right)\mathrm{d}z\mathrm{dA}+{\int }_{A}{\int }_\frac{h}{2}^{\frac{h}{2}+{h}_{p}}{\rho }_{PZT}\left(\dot{U}\delta \dot{U}+\dot{V}\delta \dot{V}+\dot{W}\delta \dot{W}\right)\mathrm{dzdA} \\ & ={\int }_{A}{\int }_{-\frac{h}{2}-{h}_{p}}^{-\frac{h}{2}}{\rho }_{PZT}\left(\left[\dot{u}+z{\dot{\psi }}_{x}\right]\delta \left[\dot{u}+z{\dot{\psi }}_{x}\right]+\left[\dot{v}+z\dot{{\psi }_{\theta }} \right]\delta \left[\dot{v}+z\dot{{\psi }_{\theta }} \right]+\dot{w}\delta \dot{w}\right)\mathrm{d}z\mathrm{dA}+{\int }_{A}{\int }_{-\frac{h}{2}}^\frac{h}{2}\rho \left(z\right)\left(\left[\dot{u}+z{\dot{\psi }}_{x}\right]\delta \left[\dot{u}+z{\dot{\psi }}_{x}\right]+\left[\dot{v}+z\dot{{\psi }_{\theta }} \right]\delta \left[\dot{v}+z\dot{{\psi }_{\theta }} \right]+\dot{w}\delta \dot{w}\right)\mathrm{d}z\mathrm{dA}+{\int }_{A}{\int }_\frac{h}{2}^{\frac{h}{2}+{h}_{p}}{\rho }_{PZT}\left(\left[\dot{u}+z{\dot{\psi }}_{x}\right]\delta \left[\dot{u}+z{\dot{\psi }}_{x}\right]+\left[\dot{v}+z\dot{{\psi }_{\theta }} \right]\delta \left[\dot{v}+z\dot{{\psi }_{\theta }} \right]+\dot{w}\delta \dot{w}\right)\mathrm{dzd}A \\ & ={\int }_{A}\left[{I}_{0}\left(\dot{u}\delta \dot{u}+\dot{v}\delta \dot{v}+\dot{w}\delta \dot{w}\right)+{I}_{1}\left(\dot{u}\delta {\dot{\psi }}_{x}+\dot{v}\delta {\dot{\psi }}_{\theta }+{\dot{\psi }}_{x}\delta \dot{u}+{\dot{\psi }}_{\theta }\delta \dot{v}\right)+{I}_{2}\left({\dot{\psi }}_{x}\delta {\dot{\psi }}_{x}+{\dot{\psi }}_{\theta }\delta {\dot{\psi }}_{\theta }\right)\right]\mathrm{dA}\end{aligned}$$

where

16 $$\begin{aligned}{I}_{0} & ={\int }_{-\frac{h}{2}-{h}_{p}}^{-\frac{h}{2}}{\rho }_{PZT}\mathrm{d}z+{\int }_{-\frac{h}{2}}^\frac{h}{2}\rho \left(z\right)\mathrm{d}z+{\int }_\frac{h}{2}^{\frac{h}{2}+{h}_{p}}{\rho }_{PZT}\mathrm{dz}, \\ {I}_{1} & ={\int }_{-\frac{h}{2}-{h}_{p}}^{-\frac{h}{2}}{\rho }_{PZT}\mathrm{zd}z+{\int }_{-\frac{h}{2}}^\frac{h}{2}\rho \left(z\right)z\mathrm{d}z+{\int }_\frac{h}{2}^{\frac{h}{2}+{h}_{p}}{\rho }_{PZT}z\mathrm{dz}, \\ {I}_{2} & ={\int }_{-\frac{h}{2}-{h}_{p}}^{-\frac{h}{2}}{\rho }_{PZT}{z}^{2}\mathrm{d}z+{\int }_{-\frac{h}{2}}^\frac{h}{2}\rho \left(z\right){z}^{2}\mathrm{d}z+{\int }_\frac{h}{2}^{\frac{h}{2}+{h}_{p}}{\rho }_{PZT}{z}^{2}\mathrm{dz}\end{aligned}$$

in which ${\rho }_{PZT}$ denotes to the mass density for each PZ layer, and $A$ is the cross-sectional area of the sandwich FG-PZ cylindrical nanoshell. The strain energy variation is expressed as

17 $$\begin{aligned}\delta {\Gamma }_{\mathrm{s}} & ={\int }_{A}{\int }_{-\frac{h}{2}-{h}_{p}}^{-\frac{h}{2}}\left({\sigma }_{xx\mathrm{in}}{\varepsilon }_{xx}+{\sigma }_{\theta \theta \mathrm{in}}{\delta \varepsilon }_{\theta \theta }+{\tau }_{x\theta \mathrm{in}}\delta {\gamma }_{x\theta }+{\tau }_{xz\mathrm{in}}{\gamma }_{xz}+{\tau }_{\theta z\mathrm{in}}{\delta \gamma }_{\theta z}-{\mathcal{D}}_{x\mathrm{in}}{\mathcal{F}}_{x\mathrm{in}}-{\mathcal{D}}_{\theta \mathrm{in}}{\mathcal{F}}_{\theta \mathrm{in}}-{\mathcal{D}}_{\mathrm{zin}}{\mathcal{F}}_{\mathrm{zin}}\right)\mathrm{d}z\mathrm{dA}+{\int }_{A}{\int }_{-\frac{h}{2}}^\frac{h}{2}\left({\sigma }_{xx}^{\mathrm{FGM}}{\varepsilon }_{xx}+{\sigma }_{\theta \theta }^{\mathrm{FGM}}{\delta \varepsilon }_{\theta \theta }+{\tau }_{x\theta }^{\mathrm{FGM}}\delta {\gamma }_{x\theta }+{\tau }_{xz}^{\mathrm{FGM}}{\gamma }_{xz}+{\tau }_{\theta z}^{\mathrm{FGM}}{\delta \gamma }_{\theta z}\right)\mathrm{d}z\mathrm{dA} \\ & \quad +{\int }_{A}{\int }_\frac{h}{2}^{\frac{h}{2}+{h}_{p}}\left({\sigma }_{xx\mathrm{in}}{\varepsilon }_{xx}+{\sigma }_{\theta \theta \mathrm{in}}{\delta \varepsilon }_{\theta \theta }+{\tau }_{x\theta \mathrm{ex}}\delta {\gamma }_{x\theta }+{\tau }_{xz\mathrm{ex}}{\gamma }_{xz}+{\tau }_{\theta z\mathrm{ex}}{\delta \gamma }_{\theta z}-{\mathcal{D}}_{x\mathrm{in}}{\mathcal{F}}_{x\mathrm{in}}-{\mathcal{D}}_{\theta \mathrm{ex}}{\mathcal{F}}_{\theta \mathrm{ex}}-{\mathcal{D}}_{\mathrm{zex}}{\mathcal{F}}_{\mathrm{zex}}\right)\mathrm{dzdA} \end{aligned}$$

It is notable that the only external forces acting on the nanoshell are the incoming sound pressure and the returned sound pressure in the external acoustic medium as well as the transferred sound pressure inside shell. Consequently, the variation of the work done by external forces is presented as

18 $$\delta {\Gamma }_{\mathrm{f}}={\int }_{A}\Delta P\delta wdA , \Delta P=\left({p}_{1}^{\mathrm{I}}+{p}_{1}^{\mathrm{R}}\right)-({p}_{2}^{\mathrm{T}})$$

Later replacing Eqs. (15), (17) and (18) into (14) and carrying out some manipulations, the final form of vibroacoustic equations of motion is written as

19 $$\begin{aligned} & \delta u: \frac{\partial {N}_{xx}}{\partial x}+\frac{1}{R}\frac{\partial {N}_{x\theta }}{\partial \theta }={I}_{0}\frac{{\partial }^{2}u}{\partial {t}^{2}}+{I}_{1}\frac{{\partial }^{2}{\psi }_{x}}{\partial {t}^{2}}, \\ & \delta v: \frac{\partial {N}_{x\theta }}{\partial x}+\frac{1}{R}\frac{\partial {N}_{\theta \theta }}{\partial \theta }+\frac{{Q}_{\theta z}}{R}={I}_{0}\frac{{\partial }^{2}v}{\partial {t}^{2}}+{I}_{1}\frac{{\partial }^{2}{\psi }_{\theta }}{\partial {t}^{2}}, \\ & \delta w: \frac{\partial {Q}_{xz}}{\partial x}+\frac{1}{R}\frac{\partial {Q}_{\theta z}}{\partial \theta }-\frac{{N}_{\theta \theta }}{R}={I}_{01}\frac{{\partial }^{2}w}{\partial {t}^{2}}-\Delta P, \\ & \delta {\psi }_{x}: \frac{\partial {M}_{xx}}{\partial x}+\frac{1}{R}\frac{\partial {M}_{x\theta }}{\partial \theta }-{Q}_{xz}={I}_{1}\frac{{\partial }^{2}u}{\partial {t}^{2}}+{I}_{2}\frac{{\partial }^{2}{\psi }_{x}}{\partial {t}^{2}}, \\ & \delta {\psi }_{\theta }: \frac{1}{R}\frac{\partial {M}_{\theta \theta }}{\partial \theta }+\frac{\partial {M}_{x\theta }}{\partial x}-{Q}_{\theta z}={I}_{1}\frac{{\partial }^{2}v}{\partial {t}^{2}}+{I}_{2}\frac{{\partial }^{2}{\psi }_{\theta }}{\partial {t}^{2}}, \\ & \delta {\phi }_{in}: \frac{\partial {p}_{xin}}{\partial x}+\frac{\partial {p}_{\theta in}}{\partial \theta }-{p}_{zin}=0, \\ & \delta {\phi }_{ex}: \frac{\partial {p}_{xex}}{\partial x}+\frac{\partial {p}_{\theta ex}}{\partial \theta }-{p}_{zex}=0, \end{aligned}$$

where the resultant axial forces (${N}_{xx}, {N}_{x\theta },{N}_{\theta \theta })$, bending moments (${M}_{xx}, {M}_{x\theta },{M}_{\theta \theta }$), and transverse shear forces (${Q}_{xz},{Q}_{\theta z}$) are provided in Appendix A. Finally, substituting equation (A) (with respect to Eqs. (7) and (9–10)) into Eq. (19), the size-dependent equilibrium equations in terms of displacement is derived and detailed in Appendix B.

### Fluid/structure compatibility conditions

Another noteworthy point is the lack of boundary conditions on the $z$ axis as the cylindrical shell length is practically infinite in this case. In contrast, there is indeed a coupling along the $r$ axis between the acoustic and structural domains, as in^86,87^

20 $$\begin{aligned} {\left.\frac{\partial }{\partial r}\left({p}_{1}^{\mathrm{I}}+{p}_{1}^{\mathrm{R}}\right)\right|}_{r=R} & =-\rho {\left(\frac{\partial }{\partial t}+V.\nabla \right)}^{2}w, \\ {\left.\frac{\partial }{\partial r}{p}_{2}^{\mathrm{T}}\right|}_{r=R} & =-\rho \frac{{\partial }^{2}w}{\partial {t}^{2}},\end{aligned}$$

Finally, substituting Eqs. (3), (6) and (12) in the governing (B1) –(B7), and Eq. (20), after some manipulation, leads to the equilibrium equations in a $9\times 9$ matrix format as

21 $$\left[\begin{array}{ccccccccc}0& 0& {K}_{\mathrm{1,3}}& {K}_{\mathrm{1,4}}& {K}_{\mathrm{1,5}}& {K}_{\mathrm{1,6}}& {K}_{\mathrm{1,7}}& {K}_{\mathrm{1,8}}& {K}_{\mathrm{1,9}}\\ 0& 0& {K}_{\mathrm{2,3}}& {K}_{\mathrm{2,4}}& {K}_{\mathrm{2,5}}& {K}_{\mathrm{2,6}}& {K}_{\mathrm{2,7}}& {K}_{\mathrm{2,8}}& {K}_{\mathrm{2,9}}\\ {K}_{\mathrm{3,1}}& {K}_{\mathrm{3,2}}& {K}_{\mathrm{3,3}}& {K}_{\mathrm{3,4}}& {K}_{\mathrm{3,5}}& {K}_{\mathrm{3,6}}& {K}_{\mathrm{3,7}}& {K}_{\mathrm{3,8}}& {K}_{\mathrm{3,9}}\\ 0& 0& {K}_{\mathrm{4,3}}& {K}_{\mathrm{4,4}}& {K}_{\mathrm{4,5}}& {K}_{\mathrm{4,6}}& {K}_{\mathrm{4,7}}& {K}_{\mathrm{4,8}}& {K}_{\mathrm{4,9}}\\ 0& 0& {K}_{\mathrm{5,3}}& {K}_{\mathrm{5,4}}& {K}_{\mathrm{5,5}}& {K}_{\mathrm{5,6}}& {K}_{\mathrm{5,7}}& {K}_{\mathrm{5,8}}& {K}_{\mathrm{5,9}}\\ 0& 0& {K}_{\mathrm{6,3}}& {K}_{\mathrm{6,4}}& {K}_{\mathrm{6,5}}& {K}_{\mathrm{6,6}}& {K}_{\mathrm{6,7}}& {K}_{\mathrm{6,8}}& 0\\ 0& 0& {K}_{\mathrm{7,3}}& {K}_{\mathrm{7,4}}& {K}_{\mathrm{7,5}}& {K}_{\mathrm{7,6}}& {K}_{\mathrm{7,7}}& 0& {K}_{\mathrm{7,9}}\\ {K}_{\mathrm{8,1}}& 0& 0& 0& {K}_{\mathrm{8,5}}& 0& 0& 0& 0\\ 0& {K}_{\mathrm{9,2}}& 0& 0& {K}_{\mathrm{9,5}}& 0& 0& 0& 0\end{array}\right]\left\{\begin{array}{c}{\tilde{P }}_{1n}^{\mathrm{R}}\\ {\tilde{P }}_{2n}^{\mathrm{T}}\\ \tilde{u }\\ \tilde{v }\\ {\tilde{w }}_{1}\\ {\stackrel{\sim }{\psi }}_{x}\\ {\stackrel{\sim }{\psi }}_{\theta }\\ {\stackrel{\sim }{\phi }}_{in}\\ {\stackrel{\sim }{\phi }}_{ex}\end{array}\right\}=\left\{\begin{array}{c}0\\ 0\\ {f}_{3}\\ 0\\ 0\\ 0\\ \begin{array}{c}0\\ {f}_{8}\\ 0\end{array}\end{array}\right\},$$

where terms ${K}_{i,j}$, ${f}_{3},$ and ${f}_{10}$ are stated in Appendix C.

## STL factor

Here, it is necessary to define the transmission coefficient $(\tau )$ which is the quotient obtained by dividing the transmitted $({\Pi }^{\mathrm{tr}}$) to incident $({\Pi }^{\mathrm{inc}})$ acoustic powers. STL is defined as a logarithmic ratio of the transmission coefficient as

22 $$\begin{aligned}\mathrm{STL} & =10\mathrm{log}\frac{1}{\tau }, \\ \tau & =\frac{{\Pi }^{\mathrm{tr}}}{{\Pi }^{\mathrm{inc}}}, \\ {\Pi }^{\mathrm{tr}} & =\sum_{n}^{\infty }\frac{\pi R}{{\varepsilon }_{n}}\mathrm{Re}\left[{\tilde{P }}_{2n}^{\mathrm{T}}{H}_{n}^{\left(1\right)}\left({k}_{2r}R\right){\left(\mathrm{i}\omega \tilde{w }\right)}^{*}\right], \\ {\Pi }^{\mathrm{inc}} & =\frac{R{{p}_{0}}^{2}}{\rho c}\mathrm{cos}\alpha \end{aligned}$$

where the superscript “*” and Re[.] indicate the complex conjugate and the real part of the argument.

## Numerical results

In this section, the correctness of the presented approach is shown here via a set of validations and simplifying assumptions, then the main outcomes are discussed.

### Mode convergence diagram

**Figure 2:**
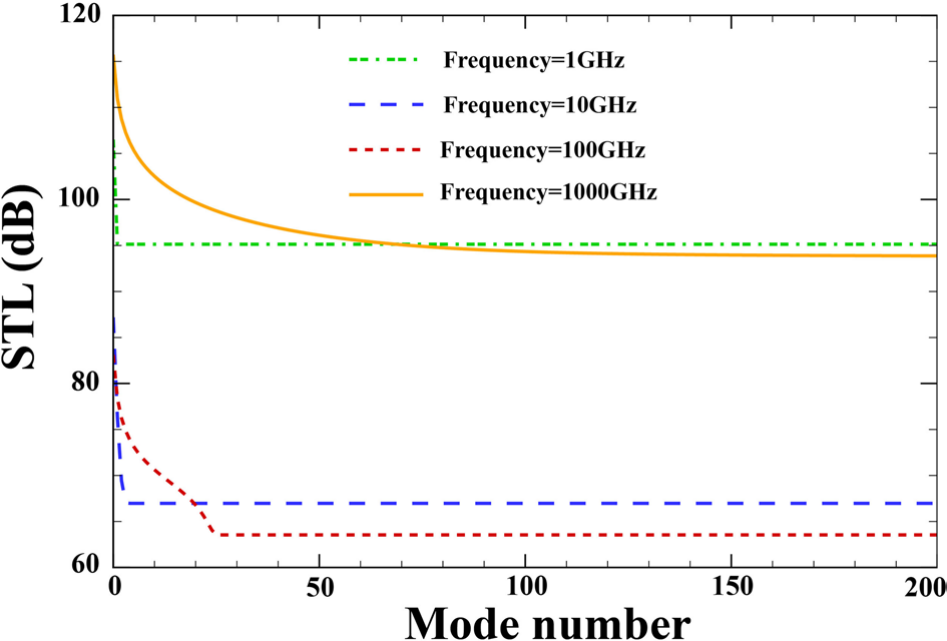
Mode convergence diagram.

Since the expansions used for the incoming and outgoing acoustic pressures and the displacement fields have infinite number of modes, the convergence of results for the STL for certain excitation frequencies at the incident angle of $\alpha ={45}{^\circ }$ is shown here. As noted in Fig. 2, with increasing excitation frequency, a higher number of modes is suggested to attain acceptable convergence. Table 1^64,71,93,94^ lists the mechanical, geometric and acoustic properties used in this analysis. Furthermore, $R=15 \; \mathrm{nm} , \; h=0.04 \; \mathrm{nm}, \; {h}_{p}=0.02 \; \mathrm{mm}, \lambda =1, \; {p}_{0}=1\; \mathrm{Pa}, \; {\phi }_{0}=0, {e}_{0}a=0,l=0 , M=0$.

**Table 1:** Material properties of the sandwich FG-PZ cylindrical nanoshell.

Properties (PZT layer)	$\mathrm{PZT}-4$	
Elastic (GPa)	${c}_{11}=132, {c}_{12}=71, {c}_{22}=132, {c}_{13}=73, {c}_{33}=115, {c}_{44}={c}_{55}=30.5$	
Piezoelectric ($\mathrm{C }{\mathrm{m}}^{-2}$)	${\mathrm{e}}_{31}=-4.1, {\mathrm{e}}_{32}=-4.1, {\mathrm{e}}_{24}=10.5, {\mathrm{e}}_{15}=10.5$, ${\mathrm{e}}_{33}=14.1$	
Dielectric (${10}^{-9}\mathrm{C }{\mathrm{V}}^{-1} {\mathrm{m}}^{-1}$)	${\upkappa }_{11}=5.841, {\upkappa }_{22}=5.841, {\upkappa }_{33}=7.124$	
Mass density ($\mathrm{Kg}$ ${\mathrm{m}}^{-3}$)	${\rho }_{\mathrm{m}}=7500$	

Properties (FG core)	Alumina (ceramic)	Steel (metal)
Elastic (GPa)	${\mathrm{E}}_{\mathrm{c}}=390$	$E=210$
Poisson’s ratio	${\mathrm{\vartheta }}_{\mathrm{c}}=0.24$	$\vartheta =0.3$
Mass density ($\mathrm{Kg}$ ${\mathrm{m}}^{-3}$)	${\uprho }_{\mathrm{c}}=3960$	$\rho =7800$

Properties (acoustic medium)	Air	
Sound speed ($\mathrm{m} {\mathrm{s}}^{-1}$)	$c=343$	
Mass density ($\mathrm{Kg}$ ${\mathrm{m}}^{-3}$)	$\rho =1.21$	

### Verification study

Owing to the intricacy of the developed procedure, a set of verifications are performed here to ensure the correctness of formulation. First, acoustic effects and PZ layers are assumed to be nonexistent, then the natural frequencies (Hz) of the FGM structure are obtained for certain indexes of power law model based on the present formulation. The results alongside other numerical findings in the literature (Ref.^95^) are shown in Table 2, indicating the acceptable accuracy of the derived equations.

**Table 2:** A comparative study of the natural frequencies of an FG cylindrical shell.

Power law index $(\lambda )$	Mode number ($n$)	Present	Ref.^95^
0	1	19.905	12.917
	2	31.578	31.603
	3	88.002	88.267
1	1	13.189	13.234
	2	32.267	32.418
	3	90.345	90.569
2	1	13.317	13.344
	2	32.549	32.683
	3	91.066	91.309

**Figure 3:**
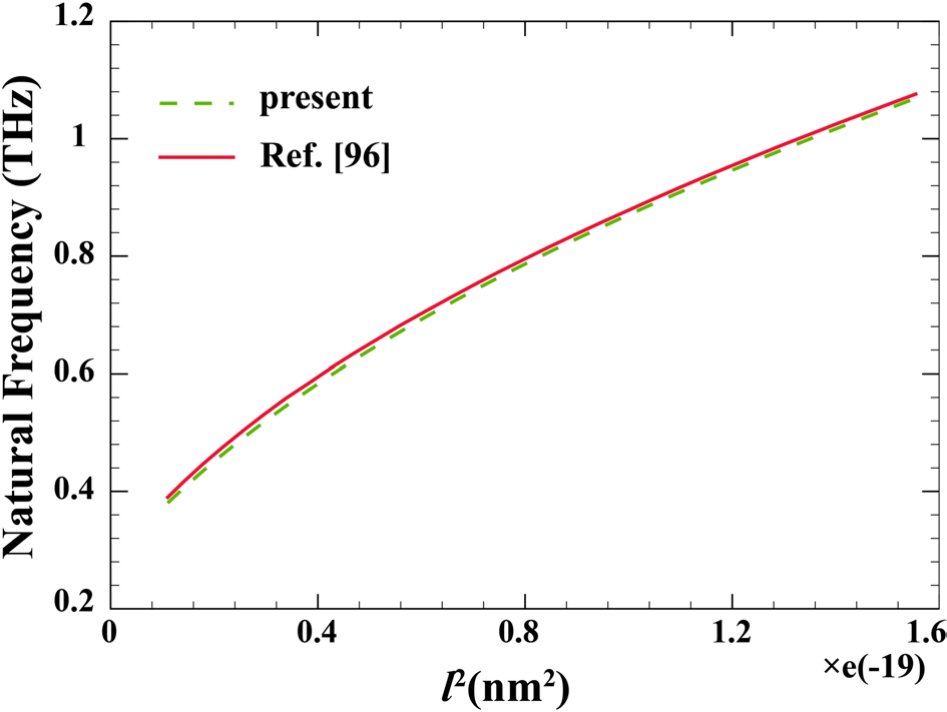
Comparison study of STL curves for a PZ nanoshell.

In the next verification study, in Fig. 3, first the FG core layer as well as acoustic effects are assumed to be absent. Then, the primary natural frequency (THz) of a PZ nanoshell ($L=R, {\left({e}_{0}a\right)}^{2}=3.3 \; {\mathrm{nm}}^{2}, {h}_{p}=0.05R$) is obtained with respect to strain gradient according to the present formulation. Comparing the obtained results with Ref.^96^ and estimating the error, once again shows that the developed formulation is capable of accommodating piezoelectric effects.

**Figure 4:**
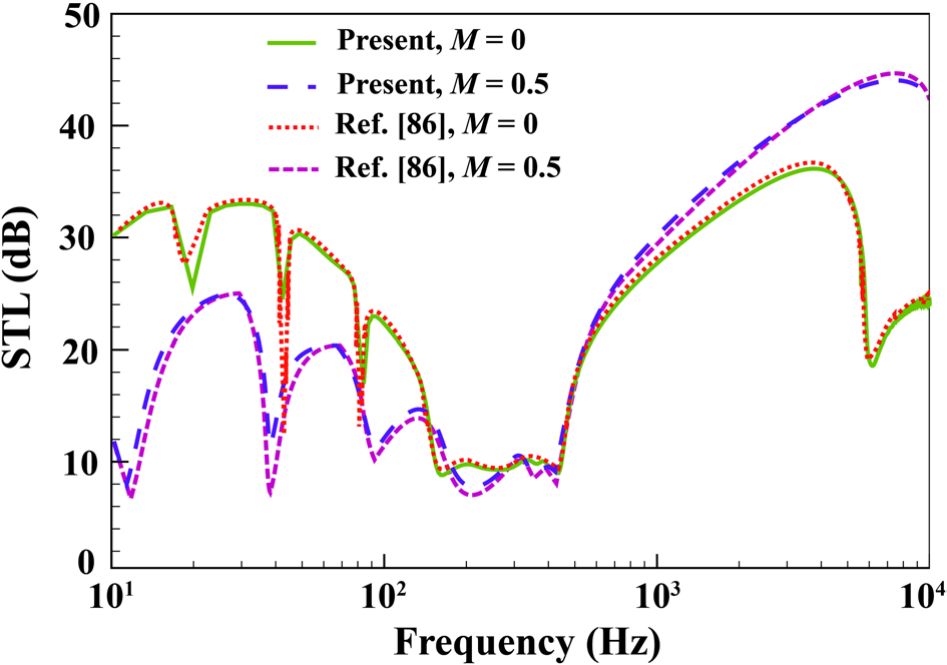
Comparison study of STL curves for single elastic cylindrical shell.

A comparison based on the classic shell theory is also presented here to further corroborate the accuracy of the described formulation. To this end, an incoming sound wave at an angle of $\alpha ={45}{^\circ }$ hits a plain cylindrical shell made of aluminum in the presence of external air/fluid flow. Under these assumptions, the STL is obtained and shown in Fig. 4 along with the numerical reports previously presented by Ref.^86^ (classical shell theory). Evidently, there is almost no tangible difference between the two sets of results.

**Figure 5:**
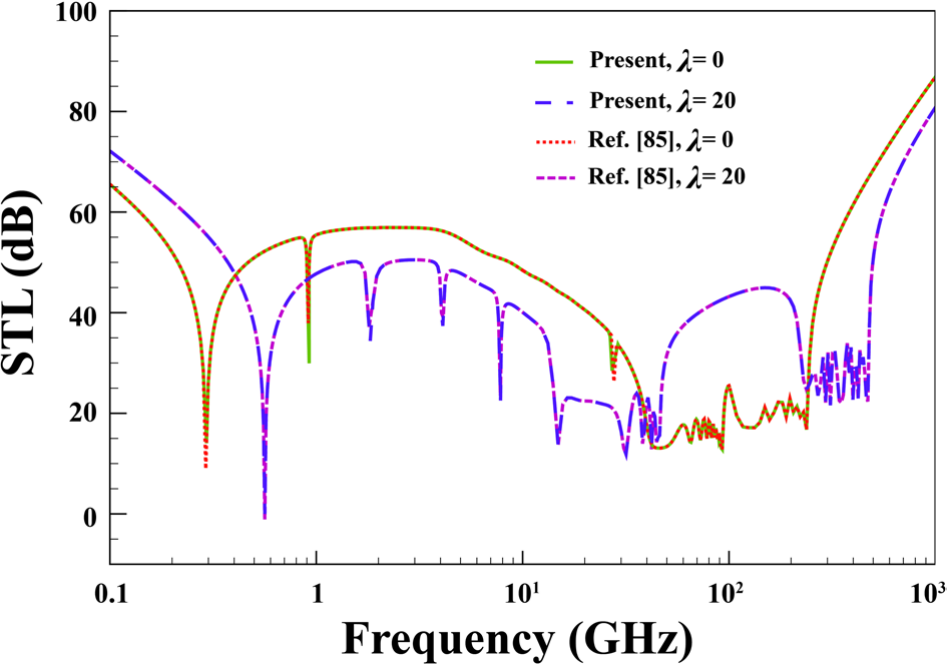
Comparison study of STL curves for an FG cylindrical nanoshell.

Finally, in Fig. 5, the STL of an FG cylindrical nanoshell ($h=0.05 \; \mathrm{nm},\; R=18.4\;\mathrm{nm}, \;\left({e}_{0}a\right)=0.03\;\mathrm{nm}, \; l=0.02\; \mathrm{nm}, \; \alpha ={45}{^\circ })$ by ignoring PZ layers, and external flow is computed based on the present formulations and compared with those reported in Ref.^85^.

### Main results

**Figure 6:**
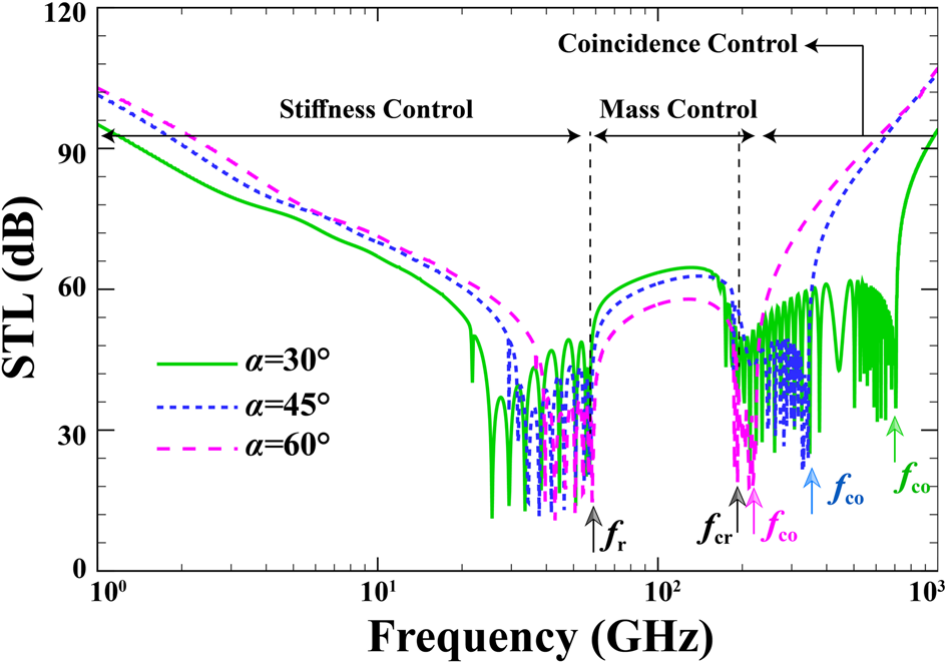
Effect of elevation angle on the variations of the STL.

Figure 6 displays the variations of STL across a sandwich FG-PZ cylindrical versus different the incidence angles over a wide frequency range (1 < f < 1000 GHz) when $R=15 \; \mathrm{nm} , \; h=0.04\; \mathrm{nm}, \; {h}_{p}=0.02\;\mathrm{mm}, \; \lambda =1, \; {p}_{0}=1\; \mathrm{Pa}, \; {\phi }_{0}=0, \; {e}_{0}a=0, \; l=0 , \; M=0$. Understanding the plots of STL is dependent on the main dips of such curves. Generally, three main dips are identified over the considered spectrum, starting with ring frequency (${\mathrm{f}}_{\mathrm{r}}$), then critical frequency (${\mathrm{f}}_{\mathrm{cr}}$), and finally coincidence frequency (${\mathrm{f}}_{\mathrm{co}}$). The ring frequency indicates the smallest value corresponding to a structural breathing mode resonance. The stiffness-controlled region lies below the ring frequency. At the critical frequency, the mode number and circumferential wave number become equal. Lastly, at the coincidence frequency, the structural wave number and the acoustic wave number become equal. While the region between the critical and coincidence is defined as mass-controlled region, any zone above the coincidence frequency is called the coincidence-controlled region. Only the coincidence frequency is affected by the angle of incoming sound wave as observed in the plots, in a way that greater incidence angles reduce the coincidence frequency. Further, an inverse relationship between the STL and the incidence angle is visible.

**Figure 7:**
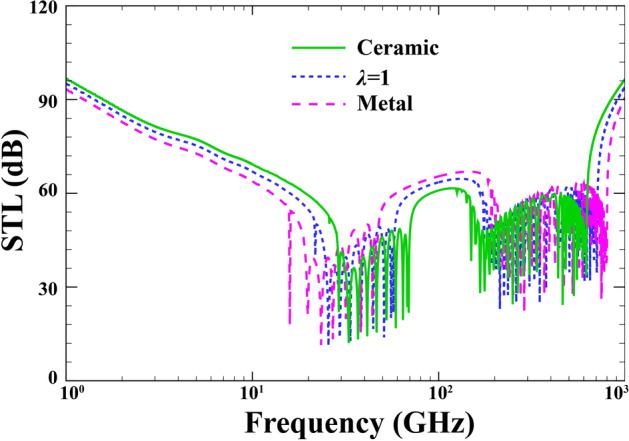
Effect of gradient index on the STL of sandwich FG-PZ nanoshell.

To investigate the effect of gradient index on the STL curves of a sandwich FG-PZ cylindrical nanoshell, Fig. 7 is presented when $R=15\; \mathrm{nm} , \; h=0.04\; \mathrm{nm}, \; {h}_{p}=0.02\;\mathrm{mm}, \; \alpha ={30}{^\circ }, \; {p}_{0}=1\; \mathrm{Pa}, \; {\phi }_{0}=0,\; {e}_{0}a=0, \; l=0 , \; M=0$. The important comments from Fig. 6 are as follows. An effective method to increase the STL at the onset of plots, i.e., the stiffness-controlled zone, is lowering the FG index. As a result, the change from ceramic to metallic state, which indicates a growth in FG index and a decrease in stiffness, reduces the sound transmission loss. In contrast, the sound transmission loss experiences a rise in the mass-controlled region with increasing FG index. It should be noted that varying the parameters of power law distribution can easily change the exact location of three characteristic frequencies.

**Figure 8:**
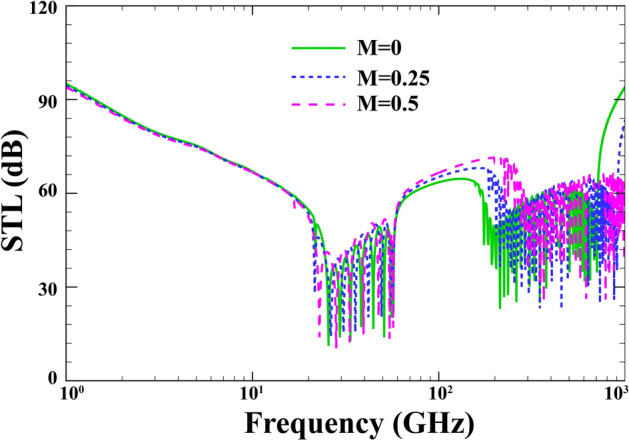
Effect of the external flow Mach number on the changes of STL.

The effect of the external flow Mach on the performance of the STL is depicted in Fig. 8 when $R=15\; \mathrm{nm} , \; h=0.04\; \mathrm{nm}, \; {h}_{p}=0.02\; \mathrm{mm}, \; \alpha ={30}{^\circ }, \; {p}_{0}=1\; \mathrm{Pa}, \; {\phi }_{0}=0, \; {e}_{0}a=0, \;l=0 , \;\lambda =1$. The radiation damping which comes into play after ${\mathrm{f}}_{\mathrm{r}}$ can augment the STL as the Mach number grows. As a result, the locations of ${\mathrm{f}}_{\mathrm{cr}}$ and ${\mathrm{f}}_{\mathrm{co}}$ are affected by varying the Mach number. This can be attributed to the total internal reflection and the acoustic radiation damping due to the altered acoustic impedance of the external convective fluid region.

**Figure 9:**
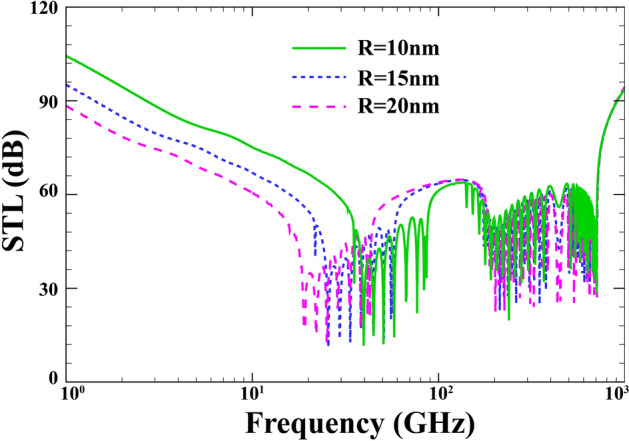
Effect of the average radius on the changes of STL.

Figure 9 shows the effect of the average radius of the sandwich FG-PZ nanoshell on the STL curve for $h=0.04 \; \mathrm{nm}, \; {h}_{p}=0.02\; \mathrm{mm},\; \alpha ={30}{^\circ },\; {p}_{0}=1\;\mathrm{Pa}, \;{\phi }_{0}=0, \;{e}_{0}a=0, \; l=0 , \; M=0$. One expects a larger reduction in STL plots with growing radius prior to ${\mathrm{f}}_{\mathrm{r}}$. In contrast, any change in the radius of the structure has no practical effect on the STL curve. At the high frequencies where the wavelengths become shorter the average radius would incorporate no effect on the STL. It should also be noted that increasing the radius of the structure decreases the ${\mathrm{f}}_{\mathrm{r}}$ value, while the ${\mathrm{f}}_{\mathrm{cr}}$ and ${\mathrm{f}}_{\mathrm{co}}$ stay unchanged.

**Figure 10:**
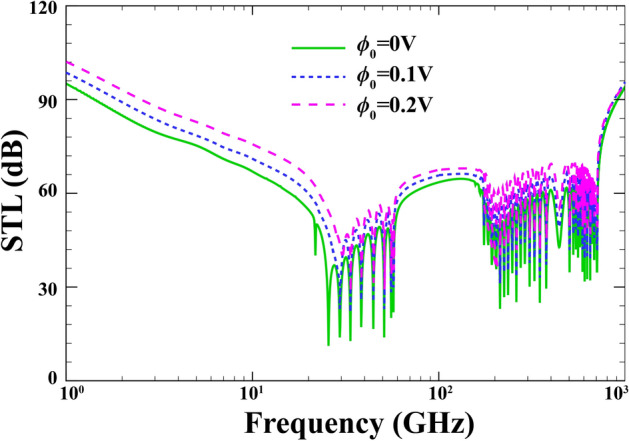
Effect of the initial electric potential on the changes of STL.

The effect of the initial electric voltage in the external PZ layer on the performance of the STL is indicated in Fig. 10 when $R=15\; \mathrm{nm} , \;h=0.04 \;\mathrm{nm}, \; {h}_{p}=0.02\;\mathrm{mm}, \; \alpha ={30}{^\circ }, \; {p}_{0}=1\;\mathrm{Pa}, \; {e}_{0}a=0, \; l=0 ,\; \lambda =1, \; M=0$. As noted from the figure, increasing the electric voltage is a suitable means to obtain better sound insulation at small frequencies, specially prior to the ring frequency. This behavior is due to the fact that by imposing positive electric voltage to the structure, tensile in-plane and compressive forces created. Nevertheless, the precise location of the three characteristic frequencies remains independent of electric potential.

**Figure 11:**
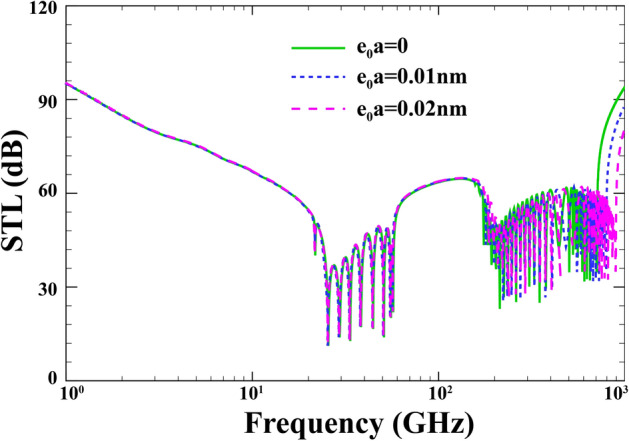
Effect of the nonlocal parameter on the changes of STL.

In Fig. 11, the effect of the nonlocal parameter on the performance of the STL is investigated when $R=15\; \mathrm{nm} , \; h=0.04\;\mathrm{nm}, \;{h}_{p}=0.02\;\mathrm{mm},\; \alpha ={30}{^\circ }, \; {p}_{0}=1\;\mathrm{Pa}, \;{\phi }_{0}=0,\; M=0,\; l=0 ,\;\lambda =1$. Some prominent researchers have formerly shown that higher values of non-local term can decrease the structural vibration frequency, which is attributed to the effect of stiffness. Interestingly, Fig. 11 shows that the STL curve is almost unaffected by the nonlocal term prior to ${\mathrm{f}}_{\mathrm{r}}$, whereas this effect becomes more prominent when the excitation frequency grows, particularly after the ${\mathrm{f}}_{\mathrm{cr}}$. Furthermore, the increasing nonlocal parameter is the reason behind the decrease in STL over the coincidence zone.

**Figure 12:**
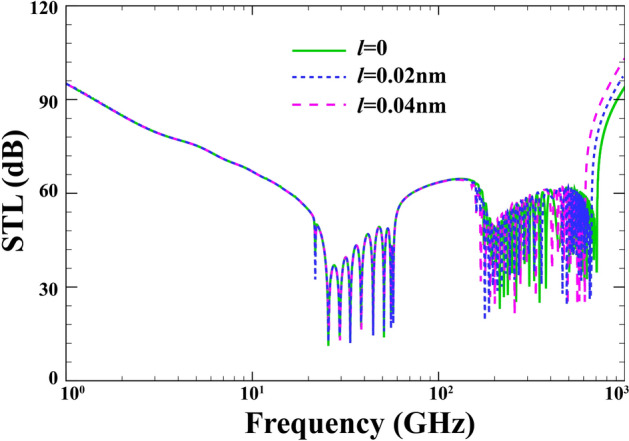
Effect of the strain gradient parameter on the changes of STL.

Figure 12 describes the variation of the STL under different values of the strain gradient parameter when $R=15\;\mathrm{nm} , \;h=0.04\;\mathrm{nm}, \;{h}_{p}=0.02\;\mathrm{mm}, \;\alpha ={30}{^\circ }, \;{p}_{0}=1\;\mathrm{Pa},\; {\phi }_{0}=0, \;{e}_{0}a=0,\;M=0 , \;\lambda =1$. As stated in earlier studies, it is expected to see a rise in vibration frequency with growing strain gradient parameter. To describe this phenomenon, one must pay attention to the stronger bounds between nanoparticle’s atoms, hence the stiffer structure. Once again, prior to ${\mathrm{f}}_{\mathrm{r}}$, the impact of strain gradient parameter on the STL curves resembles that of the nonlocal parameter. By contrast, the more rigid nature of the structure (as a result of higher strain gradient parameter) in the coincidence zone brings about a higher value of STL.

## Conclusion

The FSDT is shown to provide accurate results in acoustic problems. Accordingly, this article combines this theory with the NSGT to consider the response of PZ layers on the STL through FG nanoshell subjected to external flow. As already stated, the mechanical characteristics vary according to the power law in the thickness direction. Compatibility equations of fluid medium and cylindrical nanoshell allow us to obtain the final form of acoustic-structure equations using Hamilton’s principle. Additionally, the accuracy of this procedure is verified using a set of simulations and comparisons. The acoustic response over different frequency regions particularly the important regions of ${\mathrm{f}}_{\mathrm{r}}$, ${\mathrm{f}}_{\mathrm{cr}}$, and ${\mathrm{f}}_{\mathrm{co}}$ is also evaluated. The important results are discussed in the following.

- Increasing the electric voltage is a suitable means to obtain better sound insulation at small frequencies, specially prior to the ring frequency.
- The radiation damping which comes into play after ${\mathrm{f}}_{\mathrm{r}}$ can augment the STL as the Mach number grows.
- The change from ceramic to metallic state, which indicates a growth in FG index and a decrease in stiffness, reduces the STL. In contrast, the STL experiences a rise in the mass-controlled region with increasing FG index.
- STL curve is almost unaffected by the nonlocal term prior to ${\mathrm{f}}_{\mathrm{r}}$, whereas this effect becomes more prominent when the excitation frequency grows, particularly after the ${\mathrm{f}}_{\mathrm{cr}}$.
- Prior to ${\mathrm{f}}_{\mathrm{r}}$, the impact of strain gradient parameter on the STL curves resembles that of the nonlocal parameter. By contrast, the more rigid nature of the structure (as a result of higher strain gradient parameter) in the coincidence zone brings about a higher value of STL.

## Supplementary Information

Supplementary Information.
