# Supplementary material for: An analytical study of sound transmission loss of functionally graded sandwich cylindrical nanoshell integrated with piezoelectric layers
Source: Sci Rep. 2022 Feb 23;12:3048. doi: 10.1038/s41598-022-06905-1 (PMC8866426; doi:10.1038/s41598-022-06905-1)
Supplement: Supplementary file 1 — Supplementary Information. [file 41598_2022_6905_MOESM1_ESM.docx]

**APPENDIX A**

| $\left[ N_{xx},M_{xx} \right]=\int_{-\frac{h}{2}-h_{p}}^{-\frac{h}{2}} \sigma_{xx\mathrm{in}}\left[ 1,z \right]dz+\int_{-\frac{h}{2}}^{\frac{h}{2}} \sigma_{xx}^{\mathrm{FGM}}\left[ 1,z \right]dz+\int_{\frac{h}{2}}^{\frac{h}{2}+h_{p}} \sigma_{xx\mathrm{ex}}\left[ 1,z \right]\mathrm{dz},$ ,  $\left[ N_{x\theta},M_{x\theta} \right]=\int_{-\frac{h}{2}-h_{p}}^{-\frac{h}{2}} \tau_{x\theta\mathrm{in}}\left[ 1,z \right]dz+\int_{-\frac{h}{2}}^{\frac{h}{2}} \tau_{x\theta}^{\mathrm{FG}}\left[ 1,z \right]dz+\int_{\frac{h}{2}}^{\frac{h}{2}+h_{p}} \tau_{x\theta\mathrm{ex}}\left[ 1,z \right]\mathrm{dz}$ ,  $\left[ N_{\theta\theta},M_{\theta\theta} \right]=\int_{-\frac{h}{2}-h_{p}}^{-\frac{h}{2}} \sigma_{\theta\theta\mathrm{in}}\left[ 1,z \right]dz+\int_{-\frac{h}{2}}^{\frac{h}{2}} \sigma_{\theta\theta}^{\mathrm{FG}}\left[ 1,z \right]dz+\int_{\frac{h}{2}}^{\frac{h}{2}+h_{p}} \sigma_{\theta\theta\mathrm{ex}}\left[ 1,z \right]\mathrm{dz}$ ,  $Q_{xz}=k_{s}\left\{ \int_{-\frac{h}{2}-h_{p}}^{-\frac{h}{2}} \tau_{xz\mathrm{in}}dz+\int_{-\frac{h}{2}}^{\frac{h}{2}} \tau_{xz}^{\mathrm{FG}}dz+\int_{\frac{h}{2}}^{\frac{h}{2}+h_{p}} \tau_{xz\mathrm{ex}}\mathrm{dz} \right\}$ ,  $Q_{\theta z}=k_{s}\left\{ \int_{-\frac{h}{2}-h_{p}}^{-\frac{h}{2}} \tau_{\theta z\mathrm{in}}dz+\int_{-\frac{h}{2}}^{\frac{h}{2}} \tau_{\theta z}^{\mathrm{FG}}dz+\int_{\frac{h}{2}}^{\frac{h}{2}+h_{p}} \tau_{\theta zex}\mathrm{dz} \right\}$ ,  $p_{x\mathrm{in}}=\int_{-\frac{h}{2}-h_{p}}^{-\frac{h}{2}} \mathcal{D}_{x\mathrm{in}}\left[ \left( z+\frac{h+h_{p}}{2} \right)^{2}-\left( \frac{h_{p}}{2} \right)^{2} \right]dz,$  $p_{x\mathrm{ex}}=\int_{\frac{h}{2}}^{\frac{h}{2}+h_{p}} \mathcal{D}_{x\mathrm{ex}}\left[ \left( z-\frac{h+h_{p}}{2} \right)^{2}-\left( \frac{h_{p}}{2} \right)^{2} \right]dz$ ,  $p_{\theta\mathrm{in}}=\int_{-\frac{h}{2}-h_{p}}^{-\frac{h}{2}} \mathcal{D}_{\theta\mathrm{in}}\left[ \left( z+\frac{h+h_{p}}{2} \right)^{2}-\left( \frac{h_{p}}{2} \right)^{2} \right]dz,$ ,  $p_{\theta\mathrm{ex}}=\int_{\frac{h}{2}}^{\frac{h}{2}+h_{p}} \mathcal{D}_{\theta\mathrm{ex}}\left[ \left( z-\frac{h+h_{p}}{2} \right)^{2}-\left( \frac{h_{p}}{2} \right)^{2} \right]dz$,  $p_{z\mathrm{in}}=\int_{-\frac{h}{2}-h_{p}}^{-\frac{h}{2}} 2\mathcal{D}_{\mathrm{zin}}\left( z+\frac{h+h_{p}}{2} \right)dz$, $p_{z\mathrm{ex}}=\int_{\frac{h}{2}}^{\frac{h}{2}+h_{p}} 2\mathcal{D}_{\mathrm{zex}}\left( z-\frac{h+h_{p}}{2} \right)dz$, | (A) |
| --- | --- |

where $k_{s}$ represents the shear correction factor. This important parameter depends on the material properties, geometry, boundary conditions and loads. Generally, for a structure of rectangular cross-section, a correction factor of 5/6 yields suitable results at macro scale, although the size-dependent material characteristics at nanoscale may make this value unreliable. Nevertheless, a value of 5/6 is chosen in the rest of this study.

**APPENDIX B**

$\delta u: \left[ 1-l^{2}\nabla^{2} \right]\left\{ A_{11}\frac{\partial^{2}u}{\partial x^{2}}+B_{11}\frac{\partial^{2}\psi_{x}}{\partial x^{2}}+A_{12}\frac{1}{R}\left( \frac{\partial^{2}v}{\partial x\partial\theta}+\frac{\partial w}{\partial x} \right)+\frac{B_{12}}{R}\frac{\partial^{2}\psi_{\theta}}{\partial x\partial\theta}+F_{31}\frac{\partial\phi_{in}}{\partial x}+\bar{F}_{31}\frac{\partial\phi_{ex}}{\partial x}+\frac{A_{66}}{R}\left( \frac{\partial^{2}v}{\partial x\partial\theta}+\frac{1}{R}\frac{\partial^{2}u}{\partial\theta^{2}} \right)+\frac{B_{66}}{R}\left( \frac{\partial^{2}\psi_{\theta}}{\partial x\partial\theta}+\frac{1}{R}\frac{\partial^{2}\psi_{x}}{\partial\theta^{2}} \right) \right\}=\left[ 1-\left( e_{0}a \right)^{2}\nabla^{2} \right]\left\{ I_{0}\frac{\partial^{2}u}{\partial t^{2}}+I_{1}\frac{\partial^{2}\psi_{x}}{\partial t^{2}} \right\},$ (B1)

$\delta v: \left[ 1-l^{2}\nabla^{2} \right]\left\{ A_{66}\left( \frac{\partial^{2}v}{\partial x^{2}}+\frac{1}{R}\frac{\partial^{2}u}{\partial x\partial\theta} \right)+B_{66}\left( \frac{\partial^{2}\psi_{\theta}}{\partial x^{2}}+\frac{1}{R}\frac{\partial^{2}\psi_{x}}{\partial x\partial\theta} \right)+\frac{A_{12}}{R}\frac{\partial^{2}u}{\partial x\partial\theta}+\frac{B_{12}}{R}\frac{\partial^{2}\psi_{x}}{\partial x\partial\theta}+\frac{A_{22}}{R}\frac{1}{R}\left( \frac{\partial^{2}v}{\partial\theta^{2}}+\frac{\partial w}{\partial\theta} \right)+F_{32}\frac{\partial\phi_{in}}{\partial\theta}+\bar{F}_{32}\frac{\partial\phi_{ex}}{\partial\theta}+\frac{B_{22}}{R}\frac{1}{R}\frac{\partial^{2}\psi_{\theta}}{\partial\theta^{2}}+\frac{k_{s}}{R}A_{44}\left( \psi_{\theta}+\frac{1}{R}\frac{\partial w}{\partial\theta}-\frac{v}{R} \right)+k_{s}L_{32}\frac{\partial\phi_{in}}{\partial\theta}+k_{s}\bar{L}_{32}\frac{\partial\phi_{ex}}{\partial\theta} \right\}=\left[ 1-\left( e_{0}a \right)^{2}\nabla^{2} \right]\left\{ I_{0}\frac{\partial^{2}v}{\partial t^{2}}+I_{1}\frac{\partial^{2}\psi_{\theta}}{\partial t^{2}} \right\}$ , (B2)

$\delta w: \left[ 1-l^{2}\nabla^{2} \right]\left\{ k_{s}A_{55}\left( \frac{\partial\psi_{x}}{\partial x}+\frac{\partial^{2}w}{\partial x^{2}} \right)+k_{s}L_{31}\frac{\partial^{2}\phi_{in}}{\partial x^{2}}+k_{s}\bar{L}_{31}\frac{\partial^{2}\phi_{ex}}{\partial x^{2}}+\frac{k_{s}A_{44}}{R}\left( \frac{\partial\psi_{\theta}}{\partial\theta}+\frac{1}{R}\frac{\partial^{2}w}{\partial\theta^{2}}-\frac{1}{R}\frac{\partial v}{\partial\theta} \right)+k_{s}L_{32}\frac{\partial^{2}\phi_{in}}{\partial\theta^{2}}+k_{s}\bar{L}_{32}\frac{\partial^{2}\phi_{ex}}{\partial\theta^{2}}-\frac{A_{12}}{R}\frac{\partial u}{\partial x}-\frac{B_{12}}{R}\frac{\partial\psi_{x}}{\partial x}-\frac{A_{22}}{R}\frac{1}{R}\left( \frac{\partial v_{1}}{\partial\theta}+w \right)-\frac{B_{22}}{R}\frac{\partial\psi_{\theta}}{\partial\theta}-\frac{1}{R}F_{32}\phi_{in}-\frac{1}{R}\bar{F}_{32}\phi_{ex} \right\}=\left[ 1-\left( e_{0}a \right)^{2}\nabla^{2} \right]\left\{ I_{0}\frac{\partial^{2}w}{\partial t^{2}}-\Delta P \right\},$ (B3)

$\delta\psi_{x}: \left[ 1-l^{2}\nabla^{2} \right]\left\{ B_{11}\frac{\partial^{2}u}{\partial x^{2}}+D_{11}\frac{\partial^{2}\psi_{x}}{\partial x^{2}}+B_{12}\frac{1}{R}\left( \frac{\partial^{2}v}{\partial x\partial\theta}+\frac{\partial w}{\partial x} \right)+\frac{D_{12}}{R}\frac{\partial^{2}\psi_{\theta}}{\partial x\partial\theta}+G_{31}\frac{\partial\phi_{in}}{\partial x}+\bar{G}_{31}\frac{\partial\phi_{ex}}{\partial x}+\frac{B_{66}}{R}\left( \frac{\partial^{2}v}{\partial x\partial\theta}+\frac{1}{R}\frac{\partial^{2}u}{\partial\theta^{2}} \right)+\frac{D_{66}}{R}\left( \frac{\partial^{2}\psi_{\theta}}{\partial x\partial\theta}+\frac{1}{R}\frac{\partial^{2}\psi_{x}}{\partial\theta^{2}} \right)-k_{s}A_{55}\left( \psi_{x}+\frac{\partial w}{\partial x} \right)+k_{s}L_{31}\frac{\partial\phi_{in}}{\partial x}+k_{s}\bar{L}_{31}\frac{\partial\phi_{ex}}{\partial x} \right\}=\left[ 1-\left( e_{0}a \right)^{2}\nabla^{2} \right]\left\{ I_{1}\frac{\partial^{2}u}{\partial t^{2}}+I_{2}\frac{\partial^{2}\psi_{x}}{\partial t^{2}} \right\},$ (B4)

${\delta\psi}_{\theta}: \left[ 1-l^{2}\nabla^{2} \right]\left\{ B_{66}\left( \frac{\partial^{2}v}{\partial x^{2}}+\frac{1}{R}\frac{\partial^{2}u}{\partial x\partial\theta} \right)+D_{66}\left( \frac{\partial^{2}\psi_{\theta}}{\partial x^{2}}+\frac{1}{R}\frac{\partial^{2}\psi_{x}}{\partial x\partial\theta} \right)+\frac{B_{12}}{R}\frac{\partial^{2}u}{\partial x\partial\theta}+\frac{D_{12}}{R}\frac{\partial^{2}\psi_{x}}{\partial x\partial\theta}+\frac{B_{22}}{R}\frac{1}{R}\left( \frac{\partial^{2}v}{\partial\theta^{2}}+\frac{\partial w}{\partial\theta} \right)+\frac{d_{22}}{R}\frac{1}{R}\frac{\partial^{2}\psi_{\theta}}{\partial\theta^{2}}+\frac{1}{R}G_{32}\frac{\partial\phi_{in}}{\partial\theta}+\frac{1}{R}\bar{G}_{32}\frac{\partial\phi_{ex}}{\partial\theta}-k_{s}A_{44}\left( \psi_{\theta}+\frac{1}{R}\frac{\partial w}{\partial\theta}-\frac{v}{R} \right)-k_{s}L_{32}\frac{\partial\phi_{in}}{\partial\theta}-k_{s}L_{32}\frac{\partial\phi_{ex}}{\partial\theta} \right\}=\left[ 1-\left( e_{0}a \right)^{2}\nabla^{2} \right]\left\{ I_{1}\frac{\partial^{2}v}{\partial t^{2}}+I_{2}\frac{\partial^{2}\psi_{\theta}}{\partial t^{2}} \right\}$ , (B5)

$\delta\phi_{in}: \left[ 1-l^{2}\nabla^{2} \right]\left\{ L_{31}\left( \frac{\partial^{2}w}{\partial x^{2}}+\frac{\partial\psi_{x}}{\partial x} \right)+L_{32}\left( \frac{\partial\psi_{\theta}}{\partial\theta}+\frac{1}{R}\frac{\partial^{2}w}{\partial\theta^{2}}-\frac{1}{R}\frac{\partial v}{\partial\theta} \right)-F_{31}\frac{\partial u}{\partial x}-G_{31}\frac{\partial\psi_{x}}{\partial x}-F_{32}\left( \frac{1}{R}\frac{\partial v}{\partial\theta}+\frac{w}{R} \right)-\frac{1}{R}G_{32}\frac{\partial\psi_{\theta}}{\partial\theta}-P_{11}\frac{\partial^{2}\phi_{in}}{\partial x^{2}}-P_{22}\frac{\partial^{2}\phi_{in}}{\partial\theta^{2}}+P_{33}\phi_{in} \right\}=0,$ (B6)

$\delta\phi_{ex}: \left[ 1-l^{2}\nabla^{2} \right]\left\{ \bar{L}_{31}\left( \frac{\partial^{2}w}{\partial x^{2}}+\frac{\partial\psi_{x}}{\partial x} \right)+\bar{L}_{32}\left( \frac{\partial\psi_{\theta}}{\partial\theta}+\frac{1}{R}\frac{\partial^{2}w}{\partial\theta^{2}}-\frac{1}{R}\frac{\partial v}{\partial\theta} \right)-\bar{F}_{31}\frac{\partial u}{\partial x}-\bar{G}_{31}\frac{\partial\psi_{x}}{\partial x}-\bar{F}_{32}\left( \frac{1}{R}\frac{\partial v}{\partial\theta}+\frac{w}{R} \right)-\frac{1}{R}\bar{G}_{32}\frac{\partial\psi_{\theta}}{\partial\theta}-\bar{P}_{11}\frac{\partial^{2}\phi_{ex}}{\partial x^{2}}-\bar{P}_{22}\frac{\partial^{2}\phi_{ex}}{\partial\theta^{2}}+\bar{P}_{33}\phi_{ex} \right\}=0,$ (B7)

where

$\left[ A_{11},B_{11},D_{11} \right]=\int_{-\frac{h}{2}-h_{p}}^{-\frac{h}{2}} c_{11}\left[ 1,z,z^{2} \right]dz+\int_{-\frac{h}{2}}^{\frac{h}{2}} \frac{E(z)}{1-\vartheta^{2}}\left[ 1,z,z^{2} \right]dz+\int_{\frac{h}{2}}^{\frac{h}{2}+h_{p}} c_{11}\left[ 1,z,z^{2} \right]\mathrm{dz},$

$\left[ A_{12},B_{12},D_{12} \right]=\int_{-\frac{h}{2}-h_{p}}^{-\frac{h}{2}} c_{12}\left[ 1,z,z^{2} \right]dz+\int_{-\frac{h}{2}}^{\frac{h}{2}} \frac{\vartheta E(z)}{1-\vartheta^{2}}\left[ 1,z,z^{2} \right]dz+\int_{\frac{h}{2}}^{\frac{h}{2}+h_{p}} c_{12}\left[ 1,z,z^{2} \right]\mathrm{dz},$

$\left[ A_{22},B_{22},D_{22} \right]=\int_{-\frac{h}{2}-h_{p}}^{-\frac{h}{2}} c_{22}\left[ 1,z,z^{2} \right]dz+\int_{-\frac{h}{2}}^{\frac{h}{2}} \frac{E(z)}{1-\vartheta^{2}}\left[ 1,z,z^{2} \right]dz+\int_{\frac{h}{2}}^{\frac{h}{2}+h_{p}} c_{22}\left[ 1,z,z^{2} \right]\mathrm{dz},$

$A_{44}=\int_{-\frac{h}{2}-h_{p}}^{-\frac{h}{2}} c_{44}dz+\int_{-\frac{h}{2}}^{\frac{h}{2}} \frac{E(z)}{2\left( 1+\vartheta^{2} \right)}dz+\int_{\frac{h}{2}}^{\frac{h}{2}+h_{p}} c_{44}\mathrm{dz},$

$A_{55}=\int_{-\frac{h}{2}-h_{p}}^{-\frac{h}{2}} c_{55}dz+\int_{-\frac{h}{2}}^{\frac{h}{2}} \frac{E(z)}{2\left( 1+\vartheta^{2} \right)}dz+\int_{\frac{h}{2}}^{\frac{h}{2}+h_{p}} c_{55}\mathrm{dz},$

$\left[ A_{66},D_{66} \right]=\int_{-\frac{h}{2}-h_{p}}^{-\frac{h}{2}} c_{66}\left[ 1,z^{2} \right]dz+\int_{-\frac{h}{2}}^{\frac{h}{2}} \frac{E(z)}{2\left( 1+\vartheta^{2} \right)}\left[ 1,z^{2} \right]dz+\int_{\frac{h}{2}}^{\frac{h}{2}+h_{p}} c_{66}\left[ 1,z^{2} \right]\mathrm{dz},$

$\left[ F_{31},G_{31} \right]=\int_{-\frac{h}{2}-h_{p}}^{-\frac{h}{2}} {2e}_{31}\left[ 1,z \right]\left( z+\frac{h+h_{p}}{2} \right)dz,$ $\left[ \bar{f}_{31},\bar{g}_{31} \right]=\int_{\frac{h}{2}}^{\frac{h}{2}+h_{p}} {2e}_{31}\left[ 1,z \right]\left( z-\frac{h+h_{p}}{2} \right)dz,$

$\left[ F_{32},G_{32} \right]=\int_{-\frac{h}{2}-h_{p}}^{-\frac{h}{2}} {2e}_{32}\left[ 1,z \right]\left( z+\frac{h+h_{p}}{2} \right)dz$ , $\left[ \bar{f}_{32},\bar{g}_{32} \right]=\int_{\frac{h}{2}}^{\frac{h}{2}+h_{p}} {2e}_{32}\left[ 1,z \right]\left( z-\frac{h+h_{p}}{2} \right)dz$ ,

$L_{31}=\int_{-\frac{h}{2}-h_{p}}^{-\frac{h}{2}} e_{15}\left[ \left( z+\frac{h+h_{p}}{2} \right)^{2}-\left( \frac{h_{p}}{2} \right)^{2} \right]dz$ , $\bar{L}_{31}=\int_{\frac{h}{2}}^{\frac{h}{2}+h_{p}} e_{15}\left[ \left( z-\frac{h+h_{p}}{2} \right)^{2}-\left( \frac{h_{p}}{2} \right)^{2} \right]dz$ ,

$L_{32}=\int_{-\frac{h}{2}-h_{p}}^{-\frac{h}{2}} e_{24}\frac{1}{R+z}\left[ \left( z+\frac{h+h_{p}}{2} \right)^{2}-\left( \frac{h_{p}}{2} \right)^{2} \right]dz$ , $\bar{L}_{32}=\int_{\frac{h}{2}}^{\frac{h}{2}+h_{p}} e_{24}\frac{1}{R+z}\left[ \left( z-\frac{h+h_{p}}{2} \right)^{2}-\left( \frac{h_{p}}{2} \right)^{2} \right]dz$ ,

$P_{11}=\int_{-\frac{h}{2}-h_{p}}^{-\frac{h}{2}} k_{11}\left[ \left( z+\frac{h+h_{p}}{2} \right)^{2}-\left( \frac{h_{p}}{2} \right)^{2} \right]^{2}dz$ , $\bar{P}_{11}=\int_{\frac{h}{2}}^{\frac{h}{2}+h_{p}} k_{11}\left[ \left( z-\frac{h+h_{p}}{2} \right)^{2}-\left( \frac{h_{p}}{2} \right)^{2} \right]^{2}dz$ ,

$P_{22}=\int_{-\frac{h}{2}-h_{p}}^{-\frac{h}{2}} k_{22}\left( \frac{1}{R+z} \right)^{2}\left[ \left( z+\frac{h+h_{p}}{2} \right)^{2}-\left( \frac{h_{p}}{2} \right)^{2} \right]^{2}dz$ ,

$\bar{P}_{22}=\int_{\frac{h}{2}}^{\frac{h}{2}+h_{p}} k_{22}\left( \frac{1}{R+z} \right)^{2}\left[ \left( z-\frac{h+h_{p}}{2} \right)^{2}-\left( \frac{h_{p}}{2} \right)^{2} \right]^{2}dz$ ,

$P_{33}=\int_{-\frac{h}{2}-h_{p}}^{-\frac{h}{2}} {4k}_{33}\left( z+\frac{h+h_{p}}{2} \right)^{2}dz$ , $\bar{P}_{33}=\int_{\frac{h}{2}}^{\frac{h}{2}+h_{p}} {4k}_{33}\left( z-\frac{h+h_{p}}{2} \right)^{2}dz,$

**APPENDIX C**

$K_{1,3}=-\left\{ 1+l^{2}\left( k_{x}^{2}+\frac{n^{2}}{R^{2}} \right) \right\}\left( A_{11}k_{x}^{2}+\frac{A_{66}}{R^{2}}n^{2} \right)+\left\{ 1+\left( e_{0}a \right)^{2}\left( k_{x}^{2}+\frac{n^{2}}{R^{2}} \right) \right\}I_{0}\omega^{2}$ , $K_{1,4}=K_{2,3}=-\frac{i}{R}k_{x}n\left\{ 1+l^{2}\left( k_{x}^{2}+\frac{n^{2}}{R^{2}} \right) \right\}\left( A_{12}+A_{66} \right)$, $K_{1,5}=-K_{3,3}=-\frac{i}{R}k_{x}\left\{ 1+l^{2}\left( k_{x}^{2}+\frac{n^{2}}{R^{2}} \right) \right\}A_{12}$, $K_{1,6}=K_{4,3}=-\left\{ 1+l^{2}\left( k_{x}^{2}+\frac{n^{2}}{R^{2}} \right) \right\}\left( B_{11}k_{x}^{2}+\frac{B_{66}}{R^{2}}n^{2} \right)+\left\{ 1+\left( e_{0}a \right)^{2}\left( k_{x}^{2}+\frac{n^{2}}{R^{2}} \right) \right\}I_{1}\omega^{2}$, $K_{1,7}=K_{5,3}=-\frac{i}{R}k_{x}n\left\{ 1+l^{2}\left( k_{x}^{2}+\frac{n^{2}}{R^{2}} \right) \right\}\left( B_{12}+B_{66} \right)$, $K_{1,8}=-K_{6,3}=-ik_{x}F_{31}\left\{ 1+l^{2}\left( k_{x}^{2}+\frac{n^{2}}{R^{2}} \right) \right\}$,

$K_{1,9}=-K_{7,3}=-ik_{x}\bar{F}_{31}\left\{ 1+l^{2}\left( k_{x}^{2}+\frac{n^{2}}{R^{2}} \right) \right\}$, $K_{2,4}=-\left\{ 1+l^{2}\left( k_{x}^{2}+\frac{n^{2}}{R^{2}} \right) \right\}\left( A_{66}k_{x}^{2}+\frac{A_{22}}{R^{2}}n^{2}+\frac{k_{s}A_{44}}{R^{2}} \right)+\left\{ 1+\left( e_{0}a \right)^{2}\left( k_{x}^{2}+\frac{n^{2}}{R^{2}} \right) \right\}I_{0}\omega^{2}$ , $K_{2,5}=K_{3,4}=-\left\{ 1+l^{2}\left( k_{x}^{2}+\frac{n^{2}}{R^{2}} \right) \right\}\left( \frac{A_{22}}{R^{2}}n+\frac{k_{s}A_{44}}{R^{2}}n \right)$, $K_{2,6}=K_{4,4}=-\frac{i}{R}k_{x}n\left\{ 1+l^{2}\left( k_{x}^{2}+\frac{n^{2}}{R^{2}} \right) \right\}\left( B_{12}+B_{66} \right)$, $K_{2,7}=K_{5,4}=-\left\{ 1+l^{2}\left( k_{x}^{2}+\frac{n^{2}}{R^{2}} \right) \right\}\left( B_{66}k_{x}^{2}+\frac{B_{22}}{R^{2}}n^{2}-\frac{k_{s}A_{44}}{R} \right)+\left\{ 1+\left( e_{0}a \right)^{2}\left( k_{x}^{2}+\frac{n^{2}}{R^{2}} \right) \right\}I_{1}\omega^{2}$, $K_{2,8}=-\left\{ 1+l^{2}\left( k_{x}^{2}+\frac{n^{2}}{R^{2}} \right) \right\}\left( \frac{F_{32}}{R}n+\frac{k_{s}L_{32}}{R}n \right)$, $K_{2,9}=-\left\{ 1+l^{2}\left( k_{x}^{2}+\frac{n^{2}}{R^{2}} \right) \right\}\left( \frac{\bar{F}_{32}}{R}n+\frac{k_{s}\bar{;L}_{32}}{R}n \right)$, $K_{3,1}=\left\{ 1+\left( e_{0}a \right)^{2}\left( k_{x}^{2}+\frac{n^{2}}{R^{2}} \right) \right\}H_{n}^{\left( 2 \right)}\left( k_{1r}R \right)$, $K_{3,2}={-\left\{ 1+\left( e_{0}a \right)^{2}\left( k_{x}^{2}+\frac{n^{2}}{R^{2}} \right) \right\}H}_{n}^{\left( 1 \right)}\left( k_{2r}R \right)$, $K_{3,5}=-\left\{ 1+l^{2}\left( k_{x}^{2}+\frac{n^{2}}{R^{2}} \right) \right\}\left( k_{x}^{2}k_{s}A_{55}+\frac{k_{s}A_{44}}{R^{2}}n^{2}+\frac{A_{22}}{R^{2}} \right)+\left\{ 1+\left( e_{0}a \right)^{2}\left( k_{x}^{2}+\frac{n^{2}}{R^{2}} \right) \right\}I_{0}\omega^{2}$,

$K_{3,6}=-K_{4,5}=-\left\{ 1+l^{2}\left( k_{x}^{2}+\frac{n^{2}}{R^{2}} \right) \right\}\left( ik_{x}k_{s}A_{55}-ik_{x}\frac{B_{12}}{R} \right)$, $K_{3,7}=K_{5,5}=\left\{ 1+l^{2}\left( k_{x}^{2}+\frac{n^{2}}{R^{2}} \right) \right\}\left( \frac{k_{s}A_{44}}{R}n-\frac{B_{22}}{R^{2}}n \right),$ $K_{3,8}=-\left\{ 1+l^{2}\left( k_{x}^{2}+\frac{n^{2}}{R^{2}} \right) \right\}\left( k_{x}^{2}k_{s}L_{31}+\frac{k_{s}L_{32}n^{2}}{R}+\frac{f_{32}}{R} \right)$ , $K_{3,9}=-\left\{ 1+l^{2}\left( k_{x}^{2}+\frac{n^{2}}{R^{2}} \right) \right\}\left( k_{x}^{2}k_{s}\bar{L}_{31}+\frac{k_{s}\bar{L}_{32}n^{2}}{R}+\frac{\bar{F}_{32}}{R} \right)$, $f_{3}=-\left\{ 1+\left( e_{0}a \right)^{2}\left( k_{x}^{2}+\frac{n^{2}}{R^{2}} \right) \right\}p_{0}\varepsilon_{n}\left( -i \right)^{n}J_{n}\left( k_{1r}R \right)$, $K_{4,6}=-\left\{ 1+l^{2}\left( k_{x}^{2}+\frac{n^{2}}{R^{2}} \right) \right\}\left( k_{x}^{2}D_{11}+\frac{D_{66}}{R^{2}}n^{2}+k_{s}A_{55} \right)+\left\{ 1+\left( e_{0}a \right)^{2}\left( k_{x}^{2}+\frac{n^{2}}{R^{2}} \right) \right\}I_{2}\omega^{2}$ , $K_{4,7}=K_{5,6}=-ik_{x}\frac{1}{R}n\left\{ 1+l^{2}\left( k_{x}^{2}+\frac{n^{2}}{R^{2}} \right) \right\}\left( D_{12}+D_{66} \right)$ , $K_{4,8}=-\left\{ 1+l^{2}\left( k_{x}^{2}+\frac{n^{2}}{R^{2}} \right) \right\}\left( ik_{x}G_{31}+i{k_{x}k}_{s}L_{31} \right)$, $K_{4,9}=-\left\{ 1+l^{2}\left( k_{x}^{2}+\frac{n^{2}}{R^{2}} \right) \right\}\left( ik_{x}\bar{G}_{31}+i{k_{x}k}_{s}\bar{L}_{31} \right)$, $K_{5,7}=-\left\{ 1+l^{2}\left( k_{x}^{2}+\frac{n^{2}}{R^{2}} \right) \right\}\left( \frac{D_{22}}{R^{2}}n^{2}+k_{x}^{2}D_{66}+k_{s}A_{44} \right)+\left\{ 1+\left( e_{0}a \right)^{2}\left( k_{x}^{2}+\frac{n^{2}}{R^{2}} \right) \right\}I_{2}\omega^{2},$ $K_{5,8}=-\left\{ 1+l^{2}\left( k_{x}^{2}+\frac{n^{2}}{R^{2}} \right) \right\}\left( \frac{G_{32}}{R}n-k_{s}L_{32}n \right),$ $K_{5,9}=-\left\{ 1+l^{2}\left( k_{x}^{2}+\frac{n^{2}}{R^{2}} \right) \right\}\left( \frac{\bar{G}_{32}}{R}n-k_{s}\bar{L}_{32}n \right),$ $K_{6,4}=-\left\{ 1+l^{2}\left( k_{x}^{2}+\frac{n^{2}}{R^{2}} \right) \right\}\left( \frac{F_{32}}{R}n+\frac{L_{32}}{R}n \right)$ , $K_{6,5}=-\left\{ 1+l^{2}\left( k_{x}^{2}+\frac{n^{2}}{R^{2}} \right) \right\}\left( k_{x}^{2}L_{31}+\frac{L_{32}n^{2}}{R}+\frac{F_{32}}{R} \right)$ , $K_{6,6}=\left\{ 1+l^{2}\left( k_{x}^{2}+\frac{n^{2}}{R^{2}} \right) \right\}\left( ik_{x}G_{31}-ik_{x}L_{31} \right)$ , $K_{6,7}=-\left\{ 1+l^{2}\left( k_{x}^{2}+\frac{n^{2}}{R^{2}} \right) \right\}\left( \frac{G_{32}}{R}n-L_{32}n \right)$ , $K_{6,8}=\left\{ 1+l^{2}\left( k_{x}^{2}+\frac{n^{2}}{R^{2}} \right) \right\}\left( P_{11}k_{x}^{2}+P_{22}n^{2}+P_{33} \right)$, $K_{7,4}=-\left\{ 1+l^{2}\left( k_{x}^{2}+\frac{n^{2}}{R^{2}} \right) \right\}\left( \frac{\bar{F}_{32}}{R}n+\frac{\bar{L}_{32}}{R}n \right)$ ,

$K_{7,5}=-\left\{ 1+l^{2}\left( k_{x}^{2}+\frac{n^{2}}{R^{2}} \right) \right\}\left( k_{x}^{2}\bar{L}_{31}+\frac{\bar{L}_{32}n^{2}}{R}+\frac{\bar{F}_{32}}{R} \right)$ , $K_{7,6}=\left\{ 1+l^{2}\left( k_{x}^{2}+\frac{n^{2}}{R^{2}} \right) \right\}\left( ik_{x}\bar{G}_{31}-ik_{x}L_{31} \right)$ , $K_{7,7}=-\left\{ 1+l^{2}\left( k_{x}^{2}+\frac{n^{2}}{R^{2}} \right) \right\}\left( \frac{\bar{G}_{32}}{R}n-\bar{L}_{32}n \right)$ , $K_{7,9}=\left\{ 1+l^{2}\left( k_{x}^{2}+\frac{n^{2}}{R^{2}} \right) \right\}\left( \bar{P}_{11}k_{x}^{2}+\bar{P}_{22}n^{2}+\bar{P}_{33} \right)$, $K_{8,1}=k_{1r}{H_{n}^{\left( 2 \right)}}^{'}\left( k_{1r}R \right)$ , $K_{8,5}=-\rho\left( \omega-Vk_{x} \right)^{2}$ ,

$f_{9}=-p_{0}\varepsilon_{n}\left( -i \right)^{n}k_{1r}J_{n}^{'}\left( k_{1r}R \right)$, $K_{10,2}=k_{2r}{H_{n}^{\left( 1 \right)}}^{'}\left( k_{2r}R \right)$ , $K_{10,5}=-\rho\omega^{2}$ ,
